# Intersectionality in Digital Health Literacy and Digital Health Application Use in Germany: A MAIHDA Study by Gender, Educational Attainment, and Generation

**DOI:** 10.3390/healthcare14213750

**Published:** 2026-11-03

**Authors:** Niels Hannemann, Finn Houska, Daniel Lüdecke, Sidra Khan-Gökkaya, Cristina Ciupitu-Plath, Birgit Babitsch

**Affiliations:** 1 Department of New Public Health, Osnabrück University, 49074 Osnabrück, Germany; 2 Institute of Medical Sociology, University Medical Center Hamburg-Eppendorf (UKE), 20246 Hamburg, Germany; 3 Department of Patient and Care Management, University Medical Center Hamburg-Eppendorf (UKE), 20246 Hamburg, Germany; 4 Department of Public Health, Bastyr University, Kenmore, WA 98028, USA

**Keywords:** intersectionality, MAIHDA, health inequity, digital health applications, digital divide, digital health literacy

## Abstract

Background/Objectives: Social determinants of health (SDOHs) are linked to inequities in digital health application (DHA) use and digital health literacy (DHL), a predictor of DHA use, but few studies have examined these inequities intersectionally. This study examined intersectional differences by gender, educational attainment, and generation. Methods: Data were obtained from an online cross-sectional survey (STELLAR; *N* = 1200) in Germany. Intersectionality was assessed using Multilevel Analysis of Individual Heterogeneity and Discriminatory Accuracy (MAIHDA), with the intersections of gender, educational attainment, and generation defining strata for both outcomes. Results: Educational attainment was the strongest predictor of DHL and was associated with DHA use; gender was not significant for either. Generation was associated with DHA use, with higher odds for Generation Y and Z than for Traditionalists, but was not associated with DHL. Predicted DHL was highest among women with higher education from the Traditionalist or Generation Y group and lowest among men with basic education from Generation Z. Predicted DHA use was highest among women with higher education from Generation Z and lowest among men with basic education from the Baby Boomer generation. Additive main effects explained a greater share of the between-stratum variance for DHA use than for DHL, while the proportion of total variance attributable to strata remained limited for both outcomes. Conclusions: MAIHDA can identify population groups at greater risk of second-level digital divide-related inequities in DHL and DHA use, informing population-level measures to strengthen DHL and improve DHA use. Further research should clarify the underlying mechanisms.

## 1. Introduction

### 1.1. Digital Inequities in Healthcare

Despite an overall improvement in community health and well-being [1], health inequities tend to persist and, in some cases, widen [1,2]. In this regard, there is substantial evidence that many social determinants of health (SDOHs), such as older age, lower educational attainment, and gender-related differences, are associated with individual health status and the extent of health-related burdens and resources, such as inadequate living conditions [3,4], poorly developed health-related competencies [1], and a lower uptake of health-related services. Such outcome disparities contrast with the higher health-related burdens often experienced by these groups due to inequitable social and material conditions [5,6,7].

With the increasing digitalization of healthcare [8], a transformation is occurring, characterized by the interdependence of technological innovation and social change [9,10,11]. In general, digital health applications (DHAs) encompass mobile-based measures aimed at promoting better health, supporting lifestyle changes, and improving healthcare access in underserved or resource-deprived settings and regions [12,13,14]. DHAs are also designed to contribute to more targeted, patient-centered healthcare provision [15]. However, research suggests that DHA use differentials are rooted in SDOHs [16,17]. For example, less frequent DHA use is typically associated with lower educational attainment [18,19,20,21,22,23], higher age [18,21,24,25,26,27,28,29], and, less consistently, with male gender [30,31], suggesting that the unequal use of DHAs may exacerbate health inequities [32]. Nevertheless, the processes underlying the emergence of these differences remain insufficiently elucidated. Thus, a more comprehensive understanding of the factors shaping the use of DHAs is needed to support the development of targeted interventions that facilitate more equitable engagement with them [14].

#### Digital Divide

In general, the unequal use of digital technologies (here referred to as DHAs)—termed the digital divide [33,34]—denotes the manifestation of inequities within digital environments. It refers to the circumstance in which population groups disadvantaged by unequal social and economic conditions tend to benefit less from digital transformation measures and digital technologies [33,34,35,36]. The digital divide is determined by both structural and material factors, as well as by latent factors [37]. In order to capture the digital divide, three levels must be acknowledged. The first level of the digital divide addresses the lack of physical/material access to technologies [38]. The second level refers to individuals’ use of digital technologies as well as their abilities to use them [39,40,41], making, for example, digital health literacy (DHL) a prerequisite for participating in the digital transformation of healthcare [42,43], alongside accessible design, language accessibility, the affordability of digital infrastructure, institutional support, and trust in digital health technologies and healthcare systems [44]. DHL is considered a multidimensional construct [45,46,47,48] that refers to an individual’s ability to acquire and process health-related information [43,46,47,48]. Hence, DHL is gaining increasing attention in scientific discourse as an opportunity to counteract the digital divide [49,50], as individuals with adequate DHL are more likely to use DHAs [21,26,51,52,53,54,55,56,57,58,59]. However, lower DHL may also lead to an exacerbation of inequalities in the use of DHAs [56,60]. Although this suggests an association between DHL and DHA use, the two are separate constructs; the present study therefore examines them as two distinct second-level digital divide outcomes using separate MAIHDA models, and the hypothesized relationship in which DHL predicts DHA use is not modeled or tested here. DHL tends to be reflected in SDOH gradients. More specifically, it is not equally distributed across population groups [56], with differences tending to consolidate or even increase [60]. Research has revealed higher DHL among younger individuals and those with higher educational attainment [61,62], whereas research is more inconclusive with regard to gender-specific inequities in relation to DHL [61].

Similar patterns have also been observed in Germany. Repeated cross-sectional surveys conducted as part of the Health Literacy Survey Germany 2 (HLS-GER 2) and Health Literacy Survey Germany 3 (HLS-GER 3) in 2021 and 2025, respectively, indicate differences in digital health literacy across social and demographic characteristics. In particular, lower levels of digital health literacy were observed among individuals aged 65 years and older and among those with lower educational attainment [59,63].

In light of this, it can be assumed that not all individuals fully benefit from the advantages associated with (higher) DHL, which may contribute to the digital divide [64] as well as the expansion of health inequities [46], emphasizing the need for its assessment across different population groups [65]. The third level of the digital divide refers to conditions under which the expected benefits of using digital technologies are not effectively translated into digital participation or positive offline outcomes [40,41,66]. As illustrated, understanding the interrelationships among predictors contributing to the digital divide requires a differentiated approach. In this regard, an intersectional approach is increasingly recommended for examining digital inequity, particularly disparities in access to and the use of digital technologies [67], in order to support better digital health equity [68].

### 1.2. Intersectionality in Healthcare

The World Health Organization’s Conceptual Framework for Action on the Social Determinants of Health [69] distinguishes between structural determinants, including socioeconomic and political contexts, and intermediate determinants, such as material circumstances and properties, psychosocial factors, behavioral and biological influences, and access to health services. Structural determinants provide a broader context, while intermediate determinants translate these factors into differences in living conditions, stress levels, and healthcare access, resulting in unequal health outcomes. These two types of determinants do not operate in isolation but interact continuously. Social positions are affected by factors such as gender, ethnicity, educational attainment, and income, which simultaneously influence material and psychosocial conditions, including certain health outcomes. Hence, these determinants should not be understood merely as individual characteristics but as socially produced positions that reflect unequal distributions of resources, opportunities, exposure, and institutional responsiveness [69].

Despite the extensive body of research documenting SDOH contributions to health inequity [70], as well as the compounding effect of horizontal and vertical dimensions leading to health inequities [71,72], their intersections remain insufficiently understood [73]. This condition becomes relevant when a specific health-related outcome arising from structural inequities is generalized to an entire social group and subsequently employed as a defining characteristic of all its members, thereby neglecting potential interindividual variation in the manifestation and extent of that outcome [74]. Hence, in neglecting the intersectionality of SDOHs shaping health inequities, existing research may not sufficiently reveal their complexities, which can lead to inadequate identification and mitigation efforts [75,76], especially considering that digital health-related competencies are increasingly intersecting with established SDOHs and that the outcomes influence one another [77]. In this context, intersectional research can contribute to a more comprehensive overview and understanding of health inequities [68,72,78], which in turn can serve as a basis for designing tailored prevention programs and the establishment of targeted resource allocation, particularly for populations at risk that are facing various intersecting social, material, and institutional barriers [79,80,81].

The perspective of intersectionality was initially proposed by Crenshaw [82] to denote the intertwining of social characteristics such as race and gender and their simultaneous effects on the experiences of discrimination and disadvantage of African American women [82,83]. This can influence the intersection of further individual characteristics (e.g., age and socioeconomic status) in determining an individual’s social position, which in turn influences the availability and utilization of (health-related) resources [84]. In general, intersectionality assumes that individual health outcomes are not adequately explained by examining isolated SDOHs [85,86,87]. Instead, it argues that multiple dimensions of SDOH are interrelated and mutually reinforcing, influencing health through their interconnected and reciprocal effects [88,89]. In turn, these effects contribute to the emergence of health inequities [89]. This perspective provides an analytical framework for capturing health disparities and for examining how structural societal processes shape health outcomes across differentiated population groups [90], and as an approach, it is capable of enabling and supporting the development of effective interventions in contrast to generic, non-population-specific approaches to addressing inequities [87]. Thus, it is becoming imperative to incorporate intersectionality approaches for analyzing health inequities [79,91,92] to respond to the growing demand for comprehensive, in-depth analyses in healthcare [8]. Such measures enable the assessment of the cumulative effects of SDOHs on health inequities, addressing their underlying causes and contributing to their mitigation [79].

To summarize, despite evidence of social and health-related inequities in DHL and DHA use, research to date has mainly considered SDOHs in isolation. As a result, it remains unclear to what extent the intersection of SDOHs such as gender, educational attainment, and generation jointly affects DHL and DHA use. Conventional multivariable regression models primarily capture additive, average associations between individual social positions and an outcome, while fully specified interaction models become increasingly complex and difficult to interpret as several social dimensions are combined. The multilevel analysis of individual heterogeneity and discriminatory accuracy (MAIHDA) addresses these challenges by modeling individuals as nested within intersectional strata, estimating outcome differences across all strata simultaneously, and decomposing between-stratum variation into additive main effects and residual variation that may reflect intersectional effects. This approach is more parsimonious than specifying all higher-order interactions, provides a direct measure of between-stratum heterogeneity, and uses precision weighting to yield more stable estimates for strata with smaller sample sizes. MAIHDA therefore provides an analytical framework to contribute to a more comprehensive understanding of intersectional inequities in DHL and DHA use and to support the development of targeted, equitable measures so that the benefits of DHAs are accessible to broader segments of the population [91].

### 1.3. Scope and Research Questions

To our knowledge, this is the first MAIHDA study to examine intersectionality in relation to levels of DHL and DHA use. Despite growing evidence on social determinants of DHL and DHA use, these determinants have predominantly been examined in isolation, and it remains unclear how generation, educational attainment, and gender jointly shape inequities in DHL and DHA use when considered from an intersectional perspective. Based on existing evidence, we hypothesized that both higher age (Baby Boomers and Traditionalists) and basic educational attainment would be associated with lower DHL and lower DHA use, and that intersectional strata combining these characteristics would therefore be at the greatest disadvantage. For gender, no specific directional hypothesis was formulated, given the heterogeneous and inconclusive evidence regarding gender-specific differences in DHL and DHA use; we therefore expected mixed patterns in this regard. As no previous study has applied MAIHDA to these outcomes, no prior evidence exists on whether additive or multiplicative intersectional effects would predominate. We therefore refrained from a literature-based hypothesis on this point and addressed it empirically through Research Questions 1, 3, and 5. The following research questions were examined:

**Research** **Question** **1:**  *Do intersectional strata display inequities in DHL and DHA use, and to what extent are these inequities attributable to additive main effects and intersectional interaction effects?*

**Research Question** **2:**  *How does DHL differ across intersectional strata defined by gender, educational attainment, and generation (i.e., age classification), and which strata exhibit the highest and lowest DHL?*

**Research Question** **3:**  *To what extent is the variation in DHL between intersectional strata explained by additive main effects or effects attributable to intersectional interaction?*

**Research Question** **4:**  *How does DHA use differ across intersectional strata defined by gender, educational attainment, and generation (i.e.*,* age classification), and which strata exhibit the highest and lowest probabilities for DHA use?*

**Research Question** **5:**  *To what extent is the variation in DHA use between intersectional strata explained by additive main effects or effects attributable to intersectional interaction?*

## 2. Materials and Methods

### 2.1. Study Design

This analysis used data from the nationwide cross-sectional online survey “Online survey regarding the present and future use of digital health applications and the current degree of knowledge of digital health applications offered in Germany” (STELLAR). The survey was administered by Bilendi (Cologne, Germany), an external survey provider. Participants were recruited from Bilendi’s online access panel, which includes pre-registered individuals. Panel members were invited to participate via email, which outlined the study objectives, anticipated survey duration, and details of the incentive scheme. Participation was voluntary. The survey instrument was accessible on Bilendi’s online platform, mingle. Participants received compensation from the provider via an incentive-based scheme whereby points were awarded for study participation. These points could be exchanged for monetary compensation or a variety of rewards once they had been accrued. Anonymity and data protection were assured at all times in accordance with the General Data Protection Regulation (GDPR).

The composition of the quota sample corresponded to the distribution of age, gender, and residence in a federal state (not crossed) according to the Eurostat 2021 database (reference date: 1 January 2021). Quotas for age, gender, and federal state were defined a priori based on the Eurostat 2021 database and assessed at the beginning of the survey for automated quota management. Once a quota cell was filled, further participation by individuals matching the respective characteristics was prevented, and these individuals were automatically redirected to the end of the questionnaire. Educational attainment, one of the three social determinants examined in this study, was not part of the quota criteria (see Section 2.3), so the sample should not be understood as representative of the German general population more broadly. Data collection took place from 10 March to 18 March 2022. A total of 5998 people between 18 and 74 years of age living in Germany were invited to complete an online survey. Ultimately, 1200 participants were included in the sample. The survey instrument was specifically designed to analyze DHA use. Certain components of the Health Information National Trends Survey (HINTS) questionnaires [93,94] were adapted for this survey. The study was approved by the Ethics Committee of Osnabrück University (Reference: Ethik-61/2021), ensuring that the research was conducted in accordance with the principles outlined in the Declaration of Helsinki. Additionally, this study was reported in accordance with the STROBE statement/checklist [95].

### 2.2. Operationalization

#### 2.2.1. Digital Health Literacy (Outcome Variable)

DHL was assessed using a self-developed scale [96] based on the competencies outlined in the Digital Competence Framework for Citizens (DigComp 2.1) [97], as well as the eHEALS scale [98] and the e-health literacy model [99]. The scale focuses on “information and media literacy” and “digital problem solving” [97]. Other competencies, such as “communication and collaboration” and “content creation” [97], were not operationalized in this study. The level of DHL is indicated by a sum score of 11 items rated on a five-point Likert scale (1 = fully disagree, 5 = fully agree); full item wording and response options are provided in Supplementary Table S1. Internal consistency was high (Cronbach’s α = 0.89; McDonald’s ω = 0.92). The internal structure was examined using a principal component analysis (KMO = 0.94, item-level range 0.88–0.95; Bartlett’s test: χ^2^(55) = 5753.08, *p* < 0.001; computed using check_factorstructure(performance); see Supplementary Tables S2 and S3). Parallel analysis using fa.parallel(psych) suggested retaining one component (Supplementary Figure S2), which explained 49.4% of the total variance, with item loadings ranging from 0.51 to 0.80 (Supplementary Figure S1). Items were coded such that higher values indicate higher DHL and were summed to an overall score; given the conceptual basis of item selection, the high internal consistency, and the single-component solution, aggregation into a sum score was considered justifiable.

#### 2.2.2. Use of Digital Health Applications (Outcome Variable)

DHA use was assessed using two self-reported items following this definition: whether respondents currently had a digital health application installed relating to their personal health (response options: yes; yes, but no longer used/deleted; no) and, if so, how many such applications they used regularly, defined as at least once a week, to distinguish sustained use from occasional or trial use. Respondents self-classified which of their installed applications met the given definition, rather than selecting from a predefined, independently coded list. Respondents who reported currently having and regularly using at least one application were classified as users; all others (no current application, or an application installed but no longer used) were classified as nonusers.

#### 2.2.3. Social Determinants of Health

Gender was self-reported with the response options male, female, and diverse; as no participant selected diverse, gender was analyzed as male or female. Educational attainment was assessed using the CASMIN educational classification system [100], followed by categorization into basic, medium, or higher educational attainment [101]. Generation was used instead of a common age stratification to better identify the differences resulting from the digital transformation process [102]. The generational perspective acknowledges changes in the values and motivations of different generations driven by forces such as globalization, digitalization, and changing political developments [102,103]. The following generational groups were created based on the participants’ birth year: Traditionalists (born 1922–1955), Baby Boomers (born 1956–1965), Generation X (born 1966–1980), Generation Y (born 1981–1995), and Generation Z (born in 1996 or later) [102]. This categorization may reflect broader processes of digital socialization, as individuals born after 1980, particularly members of Generation Y and Generation Z, were inherently socialized with digital technologies in comparison to older individuals [104,105].

Gender, educational attainment, and generation (age) were selected because they are central social determinants of health and because previous research links them to inequities in DHL and DHA use (see Section 1.1). The selection of these SDOHs also aimed to operationalize both the personal and positional dimensions of the Resources and Appropriation Theory [41], thereby encompassing individual-level factors and structural conditions that shape unequal access to and use of digital technologies, contributing to the digital divide. This theory represents an established theoretical framework for describing and explaining the mechanisms underlying the digital divide, particularly by accounting for how individual resources and structural positions influence patterns of technology access and use [41]. With regard to generation, its inclusion was also theoretically motivated. In the Resources and Appropriation Theory, age/generation is explicitly conceptualized as a personal category relevant to digital inequalities and the process of technology appropriation. It discusses generational differences across several dimensions of digital access, including motivation, digital skills, and usage [41]. The generation categories used in the present study represent our empirical operationalization of this broader age/generation category. Gender represents a standard sociodemographic dimension in social and health research. In the Resources and Appropriation Theory, gender is likewise conceptualized as a personal category relevant to digital inequalities [41]. Given that prior findings on gender-specific differences in DHL [61] and DHA use remain heterogeneous and inconclusive [30,31], its inclusion was considered necessary to further examine this open question within an intersectional framework. Lower educational attainment has been consistently associated with less frequent DHA use [18,19,20,21,22,23] and lower levels of DHL [59,61,62,63]. In the Resources and Appropriation Theory, educational attainment is a positional category, reflecting differential access to material and social resources relevant to technology appropriation [41]. Statistical feasibility was considered subsequently when determining the final combination of theoretically relevant dimensions and the resulting intersectional strata. The inclusion of additional social determinants, such as income, immigration status, race, employment, or socioeconomic status, while theoretically relevant to intersectional analyses of digital and health inequities, would have increased the number of intersectional strata containing fewer than 10.4 individuals, thereby compromising reliable strata estimation given the available sample size (see Section 2.3).

### 2.3. Data Preparation and Analysis

As outlined in Section 2.1, participants were recruited individually through an online access panel, without multi-stage, cluster, or household-based sampling. This recruitment structure implies that no clustering of observations beyond the intersectional strata was expected, supporting the assumption of independent level-1 observations within strata. The survey was programmed with mandatory responses for the variables used in the present analysis. Consequently, the analytical dataset contained no missing values for gender, educational attainment, generation, DHL, or DHA use. For building the intersectional strata, no prior data preparation was therefore necessary. Consequently, all combinations of gender (1 = male, 2 = female), educational attainment (1 = basic, 2 = medium, 3 = higher), and generation (1 = Traditionalists, 2 = Baby Boomer, 3 = Generation X, 4 = Generation Y, and 5 = Generation Z) could be generated, resulting in 30 intersectional strata (2 × 3 × 5 = 30). No post-stratification or survey weights were applied; all models were fitted using the unweighted quota sample as collected. The quota procedure matched the sample to the Eurostat 2021 distribution of age, gender, and federal state (see Section 2.1) only at recruitment; educational attainment, one of the three strata-defining dimensions, was not itself quota-controlled.

Each stratum received a unique ID consisting of three digits (gender/educational attainment/generation). For example, a stratum ID of 111 represents a male with basic educational attainment who belongs to the Traditionalist generation. Considering the sample size (*N* = 1200), the inclusion of additional SDOHs for building the intersectional strata would have increased the number of intersectional strata containing fewer than 10.4 individuals (see Section 4.4), which would have compromised the reliability of the strata estimates [106]. This threshold of 10.4 individuals per stratum, as reported by Evans et al. [106], reflects findings from simulation studies investigating MAIHDA models under select parameter conditions that found robust estimate accuracy at this average stratum sample size. Evans et al. [106] explicitly note that the generalizability of this finding has yet to be thoroughly investigated, and the value reflects an average sample size across strata rather than a fixed methodological requirement that each individual stratum must satisfy. Two of the 30 strata in the present analysis fell below this average (*n* = 8 and *n* = 5, respectively; see Section 3.2). These strata were retained in the main analysis, as MAIHDA is specifically designed to provide more reliable, precision-weighted estimates for small strata than conventional single-level regression approaches. To assess the robustness of the reported findings, two additional sensitivity analyses were conducted: one excluding the two strata falling below the average stratum size of 10.4 individuals reported by Evans et al. [106], resulting in 28 intersectional strata, and one replacing generation with conventional, decade-based age categories (18–29, 30–39, 40–49, 50–59, and 60–74 years). Results are reported in the Supplementary Material (28 strata: Supplementary Tables S6–S12 and Figures S7–S10; age groups: Supplementary Tables S13–S19 and Figures S11–S14). With the age classification, three of the 30 strata comprised fewer than 10 individuals (Supplementary Table S13).

For the statistical analyses, the general analytical workflow, including the definition of intersectional strata, calculation of the ICC/VPC and PCV, estimation of stratum-specific predictions, and extraction of stratum-level residuals, followed the R code provided by Makowski et al. [107] as a methodological template, employing functions from the modelbased, insight, and performance packages within the easystats ecosystem. Deviating from the glmmTMB-based implementation shown there, linear mixed-effects models were fitted using lmer(lme4) for the continuous outcome, digital health literacy (DHL), and generalized linear mixed-effects models were fitted using glmer(lme4) for the binary outcome, digital health application (DHA) use. For both the null and the full model, confidence intervals were estimated using the model_parameters(parameters) function with the default setting ci_method = NULL, which relies on a Wald t-distribution (residual degrees of freedom) for the linear DHL model and a Wald z-distribution approximation for the binary DHA model [108]. To quantify the uncertainty of the ICC/VPC estimates in the null and adjusted models, 95% confidence intervals were obtained using bootstrapping with 1000 replications. The ICC/VPC was calculated for both the null and the full model using the icc(performance) function. The tolerance for the singularity check of the random effects was set to 1 × 10^−5^. The 95% confidence interval of the ICC was estimated based on 1000 bootstrap iterations, using the bootstrap method bootMer(lme4). To estimate the PCV, a bootstrap simulation with 1000 iterations of both the null and the full model was performed using lmer(lme4) for the continuous model and glmer(lme4) with a binomial family object for the binary model. The PCV point estimate was derived from the original (non-resampled) model, while the bootstrap distribution was used to construct the corresponding 95% confidence interval using the bias-corrected interval (BCI) method. The parametric bootstrap was performed conditional on the estimated stratum-level random effects, and bootstrap replicates yielding negative or undefined PCV values were excluded. For the adjusted models, stratum-specific predicted outcome values for the 30 intersectional strata were estimated using the estimate_means(modelbased) function. Approximate 95% confidence intervals (based on standard errors derived using the delta method) were calculated for these predicted values. The fixed-effect coefficients for gender, educational attainment, and generation in the linear and logistic Models 1B represent a small set of prespecified, theoretically motivated main effects (see Section 2.2) rather than an exploratory, data-driven search across many candidate predictors; accordingly, no correction for multiple testing was applied to these coefficient-level tests. The ranking of the intersectional strata by their predicted DHL and DHA use values, by contrast, is intended as a descriptive, exploratory summary rather than a set of formally tested comparisons [106,109]. No separate correction for multiple testing was applied here either, as the shrinkage inherent to mixed models already tempers the influence of strata with small sample sizes on the ranking [109,110]. Accordingly, differences between individual strata, including the highest- and lowest-ranked strata, should not be interpreted as statistically significant unless explicitly tested. AUC values were calculated using the performance_roc() function from the performance package (part of the easystats ecosystem), which computes the receiver operating characteristic (ROC) curve based on the model-predicted probabilities against the observed binary outcome. For the calculation of AIC and BIC, compare_performance(performance) was used. By using the functions check_convergence(performance) and check_singularity(performance), convergence and singular-fit diagnostics were examined. All models were fitted using the default optimizer settings of lmer and glmer (lme4). To assess the normality assumption underlying the linear and logistic MAIHDA models, the stratum-level random effects for both outcomes were additionally extracted using the estimate_grouplevel function (modelbased) and examined via Shapiro–Wilk tests and visual inspection of their distributions (Supplementary Tables S4 and S5, Figures S3 and S5). For the adjusted logistic model, all stratum-level random effects were estimated at zero (singular fit), so their distribution could not be tested. In addition, the adjusted main models were inspected visually using the check_model function (performance): posterior predictive checks [111] compared the observed outcome with data simulated from the fitted model, influential observations were examined for the linear model, and binned residuals were examined for the logistic model (Supplementary Figures S4 and S6). All analyses were conducted in R (version 4.6.1, R Foundation for Statistical Computing, Vienna, Austria) using RStudio (version 2025.05.1+513, Posit Software, PBC, Boston, MA, USA). The complete analysis code, including the R packages used, is provided in the Supplementary Materials (File S1).

#### 2.3.1. Analysis of Intersectionality: Multilevel Analysis of Individual Heterogeneity and Discriminatory Accuracy (MAIHDA)

MAIHDA is an approach that is increasingly being applied to assess health inequities from an intersectional perspective [112,113]. Such an approach enables the assessment of the overall extent of intersectionality and of specific effects—that is, whether particular intersections deviate from what would be expected based on the characteristics of their constituents [110].

#### 2.3.2. Linear Modeling (Digital Health Literacy)

For examining health inequities from an intersectional perspective [113], MAIHDA offers a multilevel modeling approach in which the relevant social strata are specified as higher-level units [91]. Individuals are nested within intersectional strata, which are modeled as contextual units to estimate inequities across social categories. By specifying intersectional strata in this way, this approach operationalizes intersectionality by recognizing that dimensions of inequity coalesce into distinct social positions that cannot be adequately understood in isolation, while still enabling the decomposition and disentanglement of individual dimensions [106,113,114]. The null model (Equation (1)) partitions the total variance into between-stratum and within-stratum components using an unadjusted two-level random intercept model with individuals nested within intersectional strata [106,115]. Formally, *y_ij_* denotes the outcome for individual *i* (*i* = 1,…,*n_j_*) in stratum *j* (*j* = 1,…,*J*). At level 1, the outcome is modeled as a function of a stratum-specific intercept *β*_0_*_j_* and an individual-level residual *e_ij_*. At level 2, the intercept *β*_0_*_j_* is specified as the sum of the overall mean *β*_0_ and a stratum-level residual *u_j_*. The combined model yields the predicted outcome for each stratum as *β*_0_ + *u_j_* (Equation (2)). The stratum-level residual *u_j_* captures the deviation of each stratum-specific mean from the overall mean, whereas the individual-level residual *e_ij_* captures the deviation of individual outcomes from their respective stratum mean (Equation (3)) [106,110,114]. The null model (Model 1A) is specified as follows:

(1) *y_ij_* = *β*_0_*_j_* + *e_ij_*

(2) *β*_0_*_j_* = *β*_0_ + *u_j_*

(3) *y_ij_* = *β*_0_ + *u_j_* + *e_ij_*

Linear Model 1B represents an additive main-effects model in which categorical variables defining the intersectional strata (i.e., gender, generation, and educational attainment) are included as fixed effects representing the additive contributions of these dimensions. The variables correspond to the dimensions used to define the intersectional strata rather than representing independent level-2 variables. Model 1B includes dummy variables (gender, generation, and educational attainment) *x*_1_*_j_* to *x_pj_*, along with their corresponding regression coefficients *β*_1_ to *β_p_* (Equation (4)). In contrast to single-level models, these variables are modeled at the stratum level, which is why they are indexed by *j* and not by *ij*. Fixed interaction terms are not modeled; instead, the stratum-level random effect *u_j_* captures how far stratum *j* deviates from what the additive main effects alone would predict. This deviation represents residual between-stratum heterogeneity after accounting for the additive main effects. It may reflect intersectional interaction, but cannot be uniquely attributed to it. The individual-level residual retains the same interpretation as in Model 1A; it expresses the difference between the observed outcome for individual *i* in stratum *j* and the predicted mean for that stratum. *u_j_* is assumed to be normally distributed with a mean of zero and a between-stratum variance $\sigma_{u}^{2}$, which quantifies the variance between strata after controlling for the additive main effects (Equation (5)). Here, $\sigma_{u}^{2}$ denotes the between-stratum variance of the stratum-level random effects *u_j_*, whereas $\sigma_{e}^{2}$ denotes the within-stratum individual-level residual variance, as defined in Equation (6) [106,114].

(4) *y_ij_* = *β*_0_ + *β*_1_*x*_1_*_j_* + …. + *β_p_x_pj_* + *u_j_* + *e_ij_*

(5) uj~ Ν 0,σu2

(6) eij~ Ν 0,σe2

#### 2.3.3. Logistic Modeling (DHA Use)

When MAIHDA is conducted with a binary outcome *y_ij_* (1 = occurrence; 0 = non-occurrence) for an individual *i (i =* 1, …, *n_j_)* in stratum *j (j =* 1, …, *J*), effects are estimated on the log-odds scale. This model estimates the stratum-specific log-odds of the outcome, which are subsequently transformed into predicted probabilities for interpretation. This enables the calculation of an overall stratum probability, an additive main effect-based probability, and their difference. In this regard, MAIHDA is based on the Bernoulli distribution (Equation (7)), as used by Evans et al. [106], where *y_ij_* follows a Bernoulli distribution incorporating *π_ij_*. The logit function (Equation (8)) converts these probabilities to the logit scale, representing the logit of the outcome probability [106].

In logistic modeling, estimation is performed on the logit (log-odds) scale rather than directly on the probability or outcome scale [116,117]. The odds of the outcome are given by $\frac{\pi_{\mathrm{ij}}}{1-\pi_{\mathrm{ij}}}$, and the logit transformation is defined as the natural logarithm of these odds, $\log\left(\frac{\pi_{\mathrm{ij}}}{1-\pi_{\mathrm{ij}}}\right)$. The inverse logit transformation subsequently converts the linear predictor back to predicted probabilities bounded between 0 and 1. As before, the stratum-level residuals (Equation (9)) are assumed to follow a normal distribution with a mean of zero and a variance of $\sigma_{u}^{2}$. The additive main effects in the adjusted logistic model correspond to those in the linear Model 1B and are likewise included as fixed components [106].

(7) *y_ij_* ~ Bernoulli (*π_ij_*)

(8) $$\mathrm{logit}\left(\pi_{\mathrm{ij}}\right)\equiv \log\left(\frac{\pi_{\mathrm{ij}}}{1-\pi_{\mathrm{ij}}}\right)=\beta_{0}+\beta_{1}x_{1j}+\cdots +\beta_{p}x_{\mathrm{pj}}+u_{j}$$

(9) uj~ Ν 0, σu2

The VPC and PCV are calculated based on the between-stratum variance of Models 1A and 1B, as defined in Equations (5), (6) and (9). The variance partition coefficient (VPC)/intraclass correlation coefficient (ICC) (Equation (10a) for linear modeling; Equation (10b) for logistic modeling) represents the proportion of total variance in *y_ij_* occurring between strata [109]. In the null model, the VPC quantifies the proportion of total variance attributable to differences between strata and is calculated as follows [106]:

(10a) $$\mathrm{VPC}=\frac{\sigma_{u}^{2}}{\sigma_{u}^{2}+\sigma_{e}^{2}}$$

(10b) $$\mathrm{VPC}=\frac{\sigma_{u}^{2}}{\sigma_{u}^{2}+\pi^{2}/3}$$

The VPC measures the proportion of total variance attributable to between-stratum differences in the null model, while in models including additive main effects and covariates, it represents the proportion of total variance that remains attributable to between-stratum differences after accounting for these effects. Thus, it is a key measure in MAIHDA, reflecting the proportion of total variance in *y* attributable to differences between strata, which may be interpreted as capturing intersectional variation in the outcome [106,110,114]. A higher VPC indicates that the outcome varies substantially between intersectional groups but is relatively homogeneous within each group. Conversely, a lower VPC suggests greater similarity across groups but more variation within them [118]. A systematic review provides guidance on the variability of VPCs in studies addressing health-related outcomes. In this review, the reported values ranged from 0.50% to 41.90%, with a median of 5.50% [115].

As in linear models, the VPC is used to measure the share of the total variance in the outcome attributable to differences between intersectional strata [106,112]. Since the level-1 variance cannot be directly estimated in logistic models, a latent variable approach is applied, assuming a level-1 variance of *π*^2^/3 ≈ 3.29, corresponding to the variance of the standard logistic distribution. This enables the VPC to be computed analogously to linear models [106]. To evaluate refined estimates across strata outcomes and the extent to which inequities follow additive patterns, the proportional change in variance (PCV) (Equation (11)) quantifies the proportion of between-stratum variance explained by including additive main effects [109,119].

(11) PCV =σu (Model A)2−σu Model B2σu (Model A)2

The PCV, expressed as a percentage, measures how much of the between-stratum variance is explained by additive main effects. It reflects the proportion of variance reduction achieved by incorporating additive main effects into the model. A high PCV indicates that a large proportion of the between-stratum variance is explained by the main effects (i.e., gender, educational attainment, and generation), leaving a correspondingly smaller share unexplained. Conversely, a lower PCV indicates that a larger share remains unexplained by the additive main effects. This remaining between-stratum variance represents residual between-stratum heterogeneity after accounting for the additive main effects. It may reflect intersectional interaction, but cannot be uniquely attributed to it [118,120]. A systematic review provides guidance on the variability of PCVs in studies addressing health-related outcomes. Reported values ranged from 37.9% to 99.9%, with a median of 92.9% [115], and were interpreted in the logistic models analogously to the linear models [106].

Discriminatory accuracy, which is the ability of the intersectional strata to distinguish individuals with and without the outcome, is typically assessed using the ICC/VPC. Although the ICC/VPC is a common measure for multilevel models, it is less straightforward for logistic regression models, where the level-1 variance cannot be estimated and is conventionally fixed to π^2^/3 under the latent-response approach [91,121]. In this context, the VPC represents the proportion of the total latent-scale variance that is attributable to differences between social strata. The area under the receiver operating characteristic curve (AUC) quantifies the model’s discriminatory power, that is, its ability to distinguish between individuals with and without a specific outcome. The AUC can be defined as the probability that a randomly selected individual with the outcome has a higher predicted probability than one without it. In MAIHDA, where predicted probabilities depend on the intersectional stratum, the AUC represents the probability that a randomly selected individual with the outcome has a higher stratum-specific predicted probability than a randomly selected individual without the outcome [106]. AUC values range from 0.5 to 1.0 (or 50% to 100%), where 0.5 indicates no discriminatory power and 1.0 indicates perfect discrimination, with higher values reflecting better discriminatory ability [122]. In the null model, a VPC of 0% corresponds to an AUC of 50%, and a VPC of 100% corresponds to an AUC of 100% [81]. When inequities are analyzed based on broad social categories, high AUC values are not expected, as such variables inherently have limited discriminatory power. Therefore, the AUC should be interpreted within the research context and in combination with other indicators, such as the VPC. The AUC reflects the model’s ability to discriminate between individuals with and without the outcome, which is shaped mainly by the substantial within-stratum, individual-level variance that remains unexplained in both the null and additive main effects models. The PCV, by contrast, reflects how much of the between-stratum variance is explained by the additive main effects. The AUC, in turn, is expected to be nearly identical in the null and the adjusted model, because adding the strata-defining variables to a model that already includes stratum random effects makes little difference to the stratum-specific predicted probabilities. The main effects merely redistribute variance from the random to the fixed part [106].

## 3. Results

### 3.1. Sample Description

Female and male participants were almost equally represented in the sample, reflecting the gender distribution of the population living in Germany (see Table 1), with a mean age of 46.8 years (SD = 15.3). Within the generational categories, mean age decreased from the oldest to the youngest group, with narrow and non-overlapping age ranges: Traditionalists (70.3 years, SD = 2.2, range: 67–74), Baby Boomers (61.1 years, SD = 2.7, range: 57–66), Generation X (49.8 years, SD = 4.5, range: 42–56), Generation Y (34.0 years, SD = 4.4, range: 27–41), and Generation Z (22.8 years, SD = 2.5, range: 18–26). Overall, 55.3% of the participants were assigned to either Generation X or Generation Y. Medium educational attainment was the most common category at 58.5%. Basic educational attainment was reported by 14.7% of the participants. In official statistics for Germany, the lowest educational group comprised 13.3–16.8% of 25- to 64-year-olds between 2019 and 2025 [123]. Regarding the outcome variables, the mean DHL value was 41.1 (SD = 7.4), and approximately one-third (32.7%) of the study participants used DHAs. Corresponding age descriptives for the sensitivity analyses (28 intersectional strata and the alternative age-group classification) are provided in Supplementary Tables S7 and S14, respectively, and show comparable patterns.

### 3.2. Distribution of Intersectional Strata

A total of 30 intersectional strata (100%) were constituted (see Table 2). The majority of strata (93.30%) included 10 or more individuals. Two strata were smaller: women with higher educational attainment belonging to the Traditionalists (*n* = 8) and women with basic educational attainment belonging to Generation Z (*n* = 5). The largest stratum (*n* = 105) included women with medium educational attainment belonging to Generation X. To assess whether these two small strata materially influenced the results, a sensitivity analysis excluding them was additionally conducted (resulting in 28 intersectional strata; see Section 2.3 and Supplementary Material). Its results are reported at the end of Section 3.3 and Section 3.4.

### 3.3. Estimation of Intersectionality for Digital Health Literacy

In the adjusted multilevel model (Model 1B), educational attainment was the only significant predictor of DHL. Compared with individuals with basic educational attainment, those with medium educational attainment had higher DHL (*b* = 1.70, 95% CI [0.39–3.01]), and those with higher educational attainment showed an even stronger association (*b* = 2.31, 95% CI [0.84–3.78]). Gender and generation were not significantly associated with DHL (see Table 3). Descriptively, women tended to show somewhat higher DHL values than men; however, gender was not statistically significant in the adjusted model. The VPC decreased from 1.20% in the unadjusted model to 0.50% in the adjusted model, with a proportional change in variance (PCV) of 55.90%, indicating that more than half of the between-stratum differences in DHL were explained by the additive main effects of educational attainment, gender, and generation (see Table 3).

**Figure 1:**
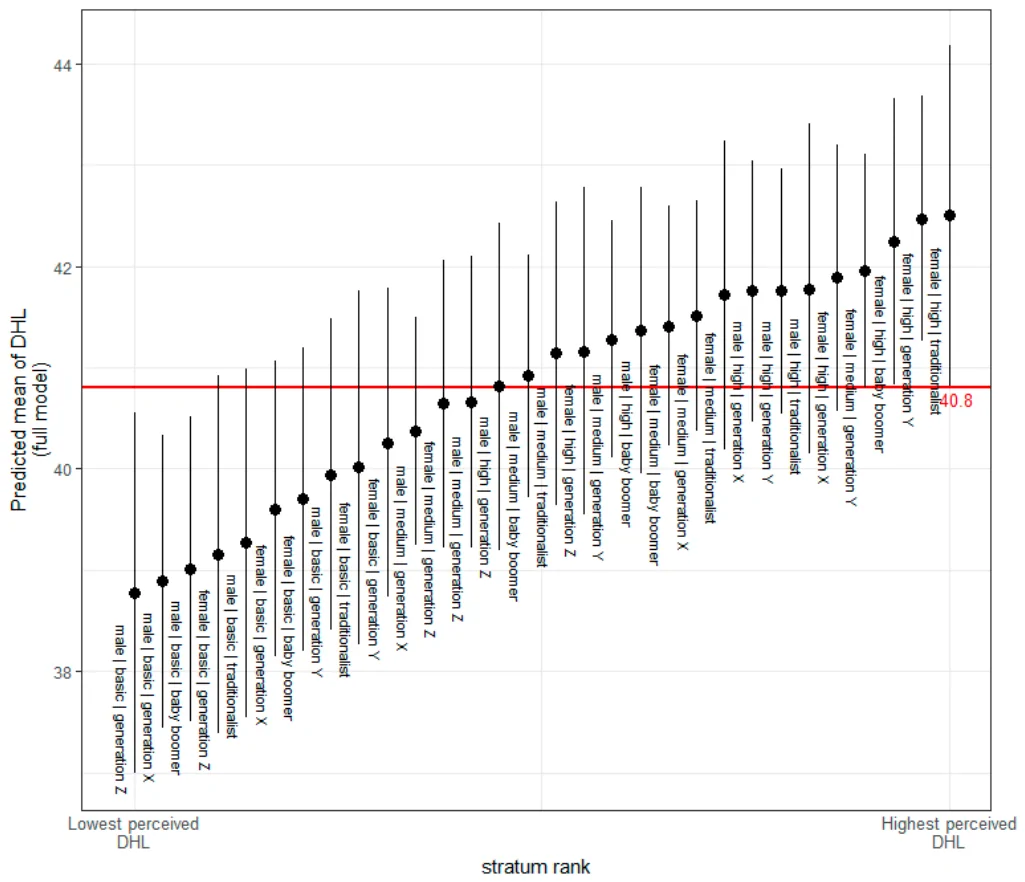
Predicted mean value of digital health literacy for all 30 strata ranked from the lowest to the highest stratum rank. Note: The red line indicates the unweighted mean of the predicted values across all 30 strata (40.8).

Based on the stratum formation selected for this analysis, the highest predicted mean DHL value was observed in the strata of women with higher educational attainment who belonged to the Traditionalist generation (predicted mean value = 42.50, 95% CI [40.80–44.20]) or to Generation Y (predicted mean value = 42.40, 95% CI [41.30–43.70]). The lowest predicted mean DHL value was found in the strata of men with basic educational attainment in Generation Z (predicted mean value = 38.80, 95% CI [37.00–40.60]), followed by men with basic educational attainment belonging to Generation X (predicted mean value = 38.90, 95% CI [37.40–40.30]) (see Table 4 and Figure 1). Convergence and singular-fit diagnostics confirmed that both the null and the adjusted linear model converged successfully without evidence of a singular fit. A Shapiro–Wilk test indicated no departure from normality for the stratum-level random effects underlying the linear MAIHDA model (*W* = 0.97, *p* = 0.42), and the two smallest strata did not stand out in this distribution, ranking 19th and 24th of 30 by estimate, indicating that the normality assumption is not disproportionately affected by these small strata. In the visual model checks, the model-predicted distribution of DHL closely resembled the observed distribution, while a small number of observations, mainly participants with very low DHL scores, were flagged as potentially influential (Supplementary Figure S4). The same diagnostics were applied to both sensitivity analyses (28 strata; alternative age classification), which likewise showed successful convergence and no evidence of a singular fit. Shapiro–Wilk tests likewise indicated no departure from normality for the linear models of both sensitivity analyses (28 strata: *W* = 0.97, *p* = 0.57; alternative age classification: *W* = 0.98, *p* = 0.73; Supplementary Tables S8 and S15, Figures S7 and S11). In the analysis with 28 strata, the VPC (1.10% and 0.60%) and the PCV (48.50%) were similar to those of the main analysis, with widely overlapping confidence intervals, and the relative ranking of the remaining strata did not change materially (Supplementary Tables S10 and S11). With the alternative age classification, educational attainment likewise remained the only significant predictor of DHL, and the VPC (2.00% and 0.70%) and the PCV (64.50%) were comparable to those of the main analysis (Supplementary Table S17).

### 3.4. Estimation of Intersectionality for the Predicted Probabilities of DHA Use

**Figure 2:**
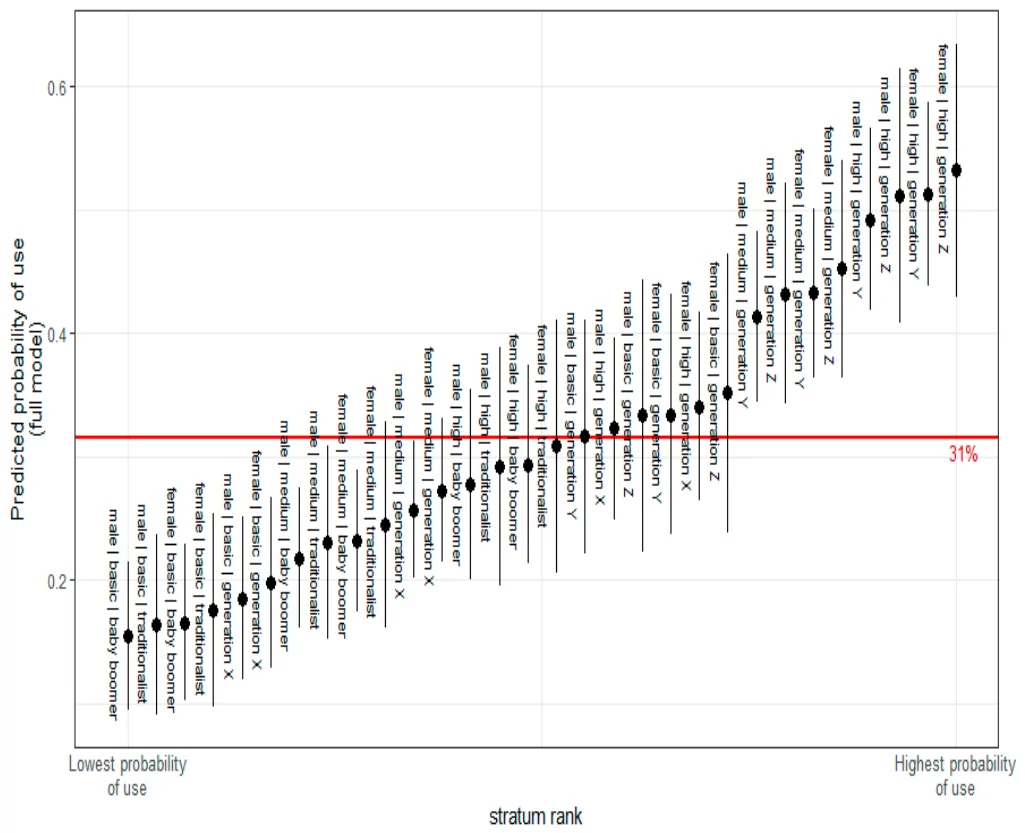
Predicted probabilities of DHA use for all 30 strata ranked from the lowest to the highest stratum rank. Note: The red line indicates the unweighted mean of the predicted probabilities across all 30 strata (31%).

For DHA use, the adjusted logistic MAIHDA model showed that higher educational attainment (OR = 2.10, 95% CI [1.36–3.24]) and medium educational attainment (OR = 1.52, 95% CI [1.02–2.27]) were associated with higher odds of DHA use. Generational effects were pronounced: Generation Y (OR = 2.35, 95% CI [1.47–3.76]) and Generation Z (OR = 2.55, 95% CI [1.50–4.32]) had higher odds of using DHAs than Traditionalists. Gender was not significant (see Table 3). Based on the stratum formation selected for this analysis, women with higher educational attainment who belonged to Generation Z were the highest-ranked stratum, with a predicted probability of DHA use of 53.20% (95% CI [42.90–63.50%]). The second-highest-ranked stratum included women with higher educational attainment who belonged to Generation Y, with a predicted probability of DHA use of 51.30% (95% CI [43.80–58.70%]). The lowest-ranked stratum included men with basic educational attainment who belonged to the Baby Boomer generation, with a predicted probability of DHA use of 15.50% (95% CI [9.40–21.50%]). Based on the ratio of predicted probabilities (53.20% versus 15.50%), the predicted probability of DHA use in the highest-ranked stratum was approximately 3.4 times higher than in the lowest-ranked stratum. The second-lowest stratum included men with basic educational attainment who belonged to the Traditionalist generation, with a predicted probability of DHA use of 16.40% (95% CI [9.10–23.70%]) (see Table 5 and Figure 2). In light of these findings, medium and higher educational attainment and belonging to Generation Y or Generation Z were associated with higher odds of DHA use, whereas the Baby Boomer generation and Generation X did not differ significantly from the Traditionalist reference group. The VPC decreased from 6.80% to 0.00% in the adjusted model, corresponding to a PCV of 100%. Convergence and singular-fit diagnostics confirmed that both the null and the adjusted logistic model converged successfully. The null model showed no evidence of a singular fit, whereas the adjusted model showed a singular fit, with the stratum-level variance estimated at zero (Supplementary Table S5, Figure S5). In the visual model checks, the model-predicted intervals included the observed counts of users and nonusers, and the binned residuals lay within the error bounds (Supplementary Figure S6). The discriminatory accuracy of the logistic MAIHDA models, assessed using the AUC, was 0.66 for Model 1A and 0.64 for Model 1B. Overall, these findings suggest that most of the observed differences between intersectional strata can be attributed to the additive main effects of educational attainment and generation (see Table 3). The same diagnostics were applied to both sensitivity analyses (28 strata; alternative age classification), which likewise showed successful convergence. As in the main analysis, the adjusted logistic model showed a singular fit in the analysis with 28 strata, whereas no singular fit occurred with the alternative age classification (Supplementary Tables S9 and S16, Figures S8 and S12). In the analysis with 28 strata, the VPC (6.90% and 0.00%) and the PCV (100%) were virtually unchanged, and the relative ranking of the remaining strata did not change materially (Supplementary Tables S10 and S12). For the model with the alternative age classification, a Shapiro–Wilk test of the stratum-level random effects indicated a departure from normality (*W* = 0.90, *p* = 0.009). As the stratum-level variance of this model was close to zero (τ_00_ = 0.01), the estimated random effects were all very small (Supplementary Table S16, Figure S12); nevertheless, the normality assumption could not be confirmed for this sensitivity model, which should be considered when interpreting its results. With the alternative age classification, the two youngest age groups (18–29 and 30–39 years) had higher odds of DHA use than the oldest group, and higher educational attainment remained significant, whereas medium educational attainment did not (OR = 1.49, 95% CI [0.99–2.25]); the VPC (6.70% and 0.20%) and the PCV (96.80%) likewise indicated predominantly additive effects (Supplementary Table S17).

## 4. Discussion

### 4.1. Principal Findings (Research Question 1)

To our knowledge, this analysis represents the first study to examine DHL and DHA use employing MAIHDA, providing initial empirical evidence for both outcomes within intersectionality research. The intersectional perspective operationalized through MAIHDA proved to be an appropriate analytical approach and demonstrated its capacity to identify disadvantaged and privileged population groups at the intersection of multiple dimensions of health inequity [79,91]. In general, the findings indicate that inequities in DHL and DHA use are structured primarily through the additive main effects of social determinants rather than through multiplicative interaction effects.

Because participation required internet access and a certain level of digital competence (see Section 4.4), DHL and DHA use are likely overestimated in this sample. The differences by social determinants are therefore likely smaller than in the general population, particularly for older and less educated individuals. The findings emphasize the relevance of the second-level digital divide, which goes beyond unequal access to digital technologies and refers to differences in skills and use patterns [39,40,41]. These inequalities could translate into unequal health benefits, a characteristic of the third-level digital divide [40,41], and thereby contribute to the persistence and reinforcement of health inequities [32,40,41]. Consequently, it needs to be emphasized that such differences in DHL and DHA use patterns should not be interpreted solely as individual-level deficits; they may also indicate unequal opportunities in developing such literacy, inequities in access to relevant digital infrastructure, and insufficient awareness of the specific needs among disadvantaged population groups to effectively engage with such digital health services.

### 4.2. Relevance of Social Determinants of Health (Research Questions 2 and 4)

Educational attainment emerged as a consistent determinant of both DHL and DHA use, supporting prior research demonstrating that lower educational attainment is associated with less frequent DHA use [18,19,20,21,22,23] and lower levels of DHL [61,62]. In contrast to our hypothesis, generational differences were not significantly associated with DHL: rather than the expected pattern of lower DHL among older generations, the highest predicted DHL was observed in a stratum of the oldest generation (Traditionalists; *n* = 8), and no significant generation effect emerged in the adjusted model. This finding also diverges from previous, non-intersectional research reporting higher DHL levels among younger individuals [61,62]. This divergence may partly reflect the online sampling design, as older participants in an online access panel are likely to be more digitally experienced than their age group in the general population (see Section 4.4), although a comparable online panel survey still found lower DHL among adults aged 65 years and older [62]. For DHA use, however, the hypothesized pattern was confirmed: members of Generation Y and Generation Z exhibited higher DHA use than Traditionalists, consistent with both our hypothesis and previous research [18,21,24,25,26,27,28,29]. These findings may be interpreted within the context of digital socialization processes. In particular, Generation Y and Generation Z [104,105] have grown up with digital technologies and therefore often possess greater technological familiarity and confidence in adopting novel technologies than older individuals [104,105]; accordingly, members of these generations appear to be more inclined to integrate digital technologies into their everyday lives, including their health-related practices. The differing patterns observed for DHL and DHA use may suggest that these outcomes are shaped by partly distinct mechanisms. While DHL appears to be more closely linked to educational resources and related competencies, DHA use may also depend on digital socialization processes, familiarity with digital technologies, and the integration of such technologies into everyday life. Accordingly, the expectation that strata combining an older generation with basic educational attainment would be at greatest disadvantage was met descriptively for DHA use, whereas the lowest predicted DHL was found in strata with basic educational attainment irrespective of generation. Gender was not significantly associated with either outcome in this study, which aligns with the inconsistent findings reported in previous research [30,31]. One possible explanation is that gender-related patterns of DHL and DHA use overlap with other determinants, such as educational attainment, which may reduce the visibility of gender-related effects; this was not examined in the present study. Nevertheless, descriptive tendencies suggested that women with higher educational attainment frequently belonged to the more advantaged strata, whereas men with basic educational attainment were represented among the more disadvantaged strata, especially concerning DHL.

### 4.3. Evaluation of Intersectionality (Research Questions 3 and 5)

A further objective of this study was to evaluate the extent to which inequities in DHL and DHA use are attributable to additive main effects versus intersectional interaction effects. The findings suggest that additive mechanisms account for an important share of between-stratum variations in both outcomes, as demonstrated by PCV [109,119] and VPC [106], particularly for DHA use. For DHL, the point estimate of the PCV (55.90%) favors an important additive contribution; however, given the very wide confidence interval (3.30–95.00%), the magnitude of this contribution remains imprecisely estimated. Nevertheless, the between-stratum variance remaining after adjustment was small (VPC = 0.50%). For DHA use, however, the stratum-level variance of the adjusted model was estimated at zero (singular fit). The PCV of 100% is therefore a boundary estimate: the additive main effects account for the between-stratum variation observed in this sample, but small interaction effects cannot be ruled out. Although the limited contribution of interaction effects may appear to challenge theoretical intersectionality tenets, the findings suggest that intersecting social positions produce distinct patterns of advantage and disadvantage in DHL and DHA use primarily through cumulative disadvantages instead of multiplicative interaction effects. Consequently, the findings demonstrate that the co-occurrence of social positions associated with unequal access to resources and opportunities remains relevant for inequities in both outcomes, even in the absence of pronounced interaction effects. However, lower VPC values should not be interpreted as indicative of weak disparities or the limited relevance of an intersectional approach. Within MAIHDA, lower VPC values are common in analyses based on SDOHs and primarily indicate substantial within-stratum heterogeneity rather than an absence of socially structured inequities [106,115]. Given this heterogeneity, together with the modest AUC values reported for DHA use, the identified strata should be understood as describing population-level patterns rather than providing a basis for individual-level classification. Thus, the value of MAIHDA resides not only in identifying interaction effects but also in making visible how combinations of social positions shape patterns of health advantage and disadvantage and in generating hypotheses about which intersectional strata may benefit from targeted prevention and health promotion strategies, which may remain obscured in conventional regression approaches. In this regard, these findings are consistent with previous MAIHDA studies demonstrating the limited discriminatory accuracy of SDOHs in predicting health-related outcomes among both individuals and population groups [106,115]. A considerable proportion of the variance remained unexplained, particularly for DHL, which may reflect its multidimensional nature [43,45,46]. For example, factors such as individuals’ general digital skills, the frequency of online health information seeking, or self-efficacy regarding health information seeking [43,47,48] may represent relevant predictors and should therefore be incorporated into future intersectional analyses. The inclusion of additional digital skills and resource-based factors would align with the World Health Organization’s Conceptual Framework for Action on the Social Determinants of Health [69], which posits that health inequities emerge through the interplay between processes of social stratification and the unequal distribution of psychosocial and non-material resources [69]. Consequently, intersectionality and its complexity may be underestimated if relevant social, psychosocial, or technology-related dimensions are omitted.

Overall, the social determinants examined were associated differently with the two outcomes, which may point to partly distinct pathways and to partly distinct intervention approaches. First, as educational attainment was associated with both outcomes, digital literacy programs and patient education for adults with lower educational attainment appear relevant for both DHL and DHA use. Second, as generation was associated with DHA use only, accessible DHA design and support from healthcare providers may help to reduce generation-related barriers among older generations. Third, the lowest predicted values were found in strata with basic educational attainment for DHL, descriptively somewhat lower among men, and in strata combining basic educational attainment with an older generation for DHA use, where disadvantages overlap; these population-level patterns may serve as a starting point for tailoring interventions, although differences between individual strata were not formally tested. Given the cross-sectional design of this study, these implications should be understood as hypothesis-generating rather than causal and not as evidence that specific strata will necessarily benefit from particular interventions; longitudinal and intervention studies are needed to determine whether targeted improvements in DHL, or approaches addressing generation-related barriers to DHA use, are in fact associated with increased and more equitable DHA use over time.

### 4.4. Limitations

Several limitations of this study need to be addressed. The intersectional strata used, which were limited to gender, educational attainment, and generation, should be interpreted as a focused selection of SDOHs rather than as an exhaustive representation of all key SDOHs for assessing intersectionality that may appear to be relevant. This operationalization was theoretically motivated (see Section 2.2) but constrained by methodological considerations, including limited sample sizes and the requirements of MAIHDA stratification. Moreover, the inclusion of additional intersectional SDOHs, especially immigration status, race, or socioeconomic status, which carry substantial risks of social exclusion and unequal health outcomes [36,69], would arguably have contributed to a more comprehensive assessment of intersectionality concerning DHL and DHA use. Although a third response option for gender (diverse) was available, it was not selected by any participant, so the analysis was restricted to male and female participants; this constitutes a further limitation, particularly within an intersectional framework. Individuals with nonbinary or diverse gender identities were therefore not represented, although they may experience distinct forms of digital and health-related inequities. Although DHL is considered a relevant predictor of DHA use [21,26,51,52,53,54,55,56,57,58,59], it was not included as a covariate or as an additional stratum-defining dimension in the logistic modeling of DHA use. On the one hand, the purpose of the analysis was to examine how the selected SDOHs structure intersectional inequities in DHL and DHA use as distinct second-level digital divide outcomes [39,40,41]. On the other hand, a further strata adjustment for this construct would have likely contributed to a higher number of intersectional strata containing fewer than 10.4 individuals per stratum, the average stratum size for which robust estimates have been reported [106]. To summarize, this selection was not merely a methodological decision but was also shaped by data availability and broader decisions about which dimensions of inequality are made visible in health research.

Although 93.30% of the intersectional strata included 10 or more individuals, the strata with fewer than 10 individuals had inherently limited explanatory power and were among the top (ID 231) and bottom (ID 215) five strata in terms of the predicted mean DHL. Hence, their relevance needs to be critically considered. However, as reported in Section 3.3 and Section 3.4, a sensitivity analysis excluding these two strata did not materially change the VPC, PCV, or the relative ranking of the remaining strata, supporting the robustness of the main findings. As the assessment of DHL was self-assessed by the study participants, it cannot be ruled out that they under- or overestimated their competencies [124]. Furthermore, the unexplained variance in DHL may be attributable to its multidimensional conceptualization [45,46], encompassing a broad set of information-processing competencies that enable individuals to access, comprehend, critically appraise, and effectively use health-related information acquired through digital technologies [46], which could not be explained sufficiently by the included SDOHs. The scale used in this study covered only selected competencies of this broader construct and summarized them in a single sum score (see Section 2.2). The DHL scale was self-developed and has not been independently validated. However, its internal consistency was high, a single component was extracted, and higher DHL scores were associated with DHA use in previous analyses of the same survey [21,54], in one of them also after adjustment for social and health-related characteristics [54]. It was not formally tested whether the scale measures the same construct comparably across gender, educational attainment, and generation (measurement invariance). Observed differences in DHL between strata could therefore partly reflect measurement non-invariance rather than genuine differences in digital health literacy and should be interpreted with caution; testing measurement invariance should be a priority for future research. DHA use was measured, as in a previous analysis of the same survey [54], as a single binary indicator of self-reported regular (at least weekly) use, which does not differentiate between specific types, purposes, intensity, or quality of use. Additionally, both DHL and DHA use levels may have been overestimated due to the selection bias inherent to the online study design, as participation required internet access and a certain level of digital literacy. Individuals with lower digital capabilities or limited access to digital resources were likely underrepresented. Consequently, the observed differences between intersectional strata should be regarded as lower-bound estimates that may underestimate the actual extent and structure of these inequities in the German general population; this should be considered when interpreting and generalizing these findings. Because detailed information on nonresponse, incomplete participation, quota-related exclusion, and quality-control procedures was unavailable, the potential for selection bias beyond the online-panel sampling frame cannot be fully quantified. The mandatory responses prevented item nonresponse but may in principle have prompted a small number of respondents to select an answer primarily to proceed. Educational attainment was not itself quota-controlled. The comparison with official statistics [123] is only approximate, because the lowest educational group is defined more narrowly there and the age ranges differ; individuals with basic educational attainment may therefore be underrepresented in the sample. Due to the cross-sectional design, no causal conclusions can be drawn, which limits the explanatory power of this analysis. As with any observational study, residual confounding by factors not captured in this dataset (e.g., income, migration background, chronic illness) cannot be ruled out. These findings may further not generalize to other countries, given differences in healthcare systems, social structures, and levels of digitalization. Moreover, as no comparable MAIHDA research on DHL and DHA use has been conducted, it is not possible to directly classify and compare our results, for example, with regard to the meaning of intersectionality and the additive effects of the included SDOHs, which therefore permits generalization to only a limited extent. More importantly, whether DHL and DHA use constitute health outcomes (e.g., morbidity or mortality) must be critically discussed. In this regard, it may be more suitable to consider them as health-related competencies and behavioral indicators that may act as intermediate determinants of health. Consequently, the comparability of findings reported in the systematic review, particularly among studies evaluating health-related outcomes [115], needs to be critically considered.

## 5. Conclusions

The application of MAIHDA provides evidence for patterns of inequities in both latent and material health-related resources [37], namely DHL and DHA use. By considering the combined effects of multiple social determinants, this approach enables the identification and quantification of disadvantaged intersectional population strata, thereby contributing to a more nuanced understanding of health inequities [80,92] and supporting efforts to advance health equity [2]. The identification of disadvantaged intersectional strata, namely population groups, further highlights the importance of interventions that address multiple dimensions of inequity [81,87]. In this regard, as exemplified by the present MAIHDA analysis, novel methodological frameworks [125,126] can enable a more comprehensive examination of intersectional inequities and the mechanisms underlying their emergence and persistence, warranting broader implementation in future research.

To increase the precision of identifying populations at greater risk of health-related burdens and inequities, as exemplified by lower DHL and less DHA use in this analysis, future research should incorporate additional social and health-related characteristics, such as immigration status, race, suffering from a chronic disease, or technology-related familiarity. This calls for the systematic and continuous collection of population-based data [92] capturing a broad spectrum of SDOHs [79], as well as additional psychosocial factors that may potentially affect DHA use, such as attitudes towards them. This requires sufficiently large samples, because additional determinants quickly lead to small intersectional strata, as was the case in the present analysis. Large population surveys should therefore record SDOHs in a differentiated manner, which depends on adequate research funding. In view of the anticipated expansion of DHA use, there is a need for interventions that may promote their equitable, appropriate, and sustained use. Given the pivotal role attributed to DHL in mitigating the digital divide [49] and its reported association with DHA use [21,26,51,52,53,54,55,56,57,58,59], strengthening DHL should be considered a public health priority, particularly among individuals with lower educational attainment, where differences in DHA use tend to persist. Addressing generational differences in technology familiarity may similarly help reduce persisting gaps in DHA use. Accordingly, intervention strategies should include user-friendly design, tailored communication, and support for digital skills development to strengthen DHA access and decrease the risk of (further) inequities within the second and third levels of the digital divide. Such interventions should prioritize equitable access to the benefits of digital applications and technologies [39,40,41] while simultaneously working to eliminate intervention-related inequities [127].

The achievement of sustainable improvements may require the implementation of regulatory and policy-level measures that establish mandatory standards for DHL promotion and program quality. Within the German healthcare system, the institutional infrastructure surrounding statutory health insurance offers a feasible platform for integrating such interventions. In addition to improving individual-level competencies, technology-related measures aimed at reducing barriers to DHA use are essential, including the development of user-friendly interfaces, the use of plain, accessible language, and the incorporation of participatory design principles. Iterative evaluation processes that integrate end-user feedback are crucial to ensure usability, acceptance, and long-term engagement. Addressing both individual-level competencies and technology-related measures in a complementary manner may foster more equitable participation in an increasingly digitalized healthcare system and expand the potential benefits of DHAs, particularly among disadvantaged population groups [128].

## Figures and Tables

**Table 1:** Sample description (*N* = 1200).

	*n*	%
Gender		
Female	602	50.2
Male	598	49.8
Generation (46.8 ^a^; 15.3 ^b^)		
Traditionalists (Year of birth: 1922–1955) (70.3 ^a^; 2.2 ^b^; Min. 67, Max. 74 ^c^)	127	10.6
Baby Boomer (Year of birth: 1956–1965) (61.1 ^a^; 2.7 ^b^; Min. 57, Max. 66 ^c^)	264	22.0
Generation X (Year of birth: 1966–1980) (49.8 ^a^; 4.5 ^b^; Min. 42, Max. 56 ^c^)	331	27.6
Generation Y (Year of birth: 1981–1995) (34.0 ^a^; 4.4 ^b^; Min. 27, Max. 41 ^c^)	333	27.8
Generation Z (Year of birth: 1996–present) (22.8 ^a^; 2.5 ^b^; Min. 18, Max. 26 ^c^)	145	12.1
Educational attainment		
Basic	176	14.7
Medium	702	58.5
Higher	322	26.8
Outcome variables		
Use of digital health applications (DHA use)	392	32.7
Digital health literacy (cont.) (DHL)	41.1 ^d^; 7.4 ^b^	-

^a^ mean age; ^b^ standard deviation; ^c^ range; ^d^ mean value.

**Table 2:** Size and distribution of intersectional strata defined by gender, educational attainment, and generation.

Size per Stratum	*n*	%
100 or more individuals	1	3.30
50 or more individuals	9	30.00
30 or more individuals	15	50.00
20 or more individuals	21	70.00
10 or more individuals	28	93.30
Fewer than 10 individuals	2	6.70

**Table 3:** Parameter estimates for linear models of DHL and logistic models of DHA use.

	Digital Health Literacy				Digital Health Application Use			
	Linear Model 1A		Linear Model 1B		Logistic Model 1A		Logistic Model 1B	
	Estimate	[95% CI]	Estimate	[95% CI]	OR	[95% CI]	OR	[95% CI]
**Fixed effects: ** **Regression coefficients**								
Intercept	41.05 ***	[40.52–41.59]	39.45 ***	[37.73–41.17]	0.44 ***	[0.35–0.56]	0.20 ***	[0.12–0.33]
**Gender**								
Male (Ref.)			-	-			-	-
Female			0.57	[−0.38–1.52]			1.08	[0.85–1.39]
**Educational attainment**								
Basic (Ref.)			-	-			-	-
Medium			1.70 *	[0.39–3.01]			1.52 *	[1.02–2.27]
Higher			2.31 **	[0.84–3.78]			2.10 ***	[1.36–3.24]
**Generation**								
Traditionalists (Ref.)			-	-			-	-
Baby Boomer			−0.29	[−1.99–1.41]			0.93	[0.56–1.54]
Generation X			−0.40	[−2.04–1.25]			1.16	[0.72–1.87]
Generation Y			0.21	[−1.45–1.87]			2.35 ***	[1.47–3.76]
Generation Z			−0.87	[−2.76–1.02]			2.55 ***	[1.50–4.32]
**Random effects: ** **Variance**								
Residual variance (σ^2^)	53.50 (SD 7.30)		53.45 (SD 7.31)		3.29 (SD 1.81)		3.29 (SD 1.81)	
Stratum-level variance (τ_00_)	0.66 (SD 0.81)		0.29 (SD 0.54)		0.24 (SD 0.49)		0.00	
VPC [95% CI]	1.20% [0.07–3.30%]		0.50% [0.05–3.00%]		6.80% [1.90–11.70%]		0.00%	
PCV [95% CI]			55.90% [3.30–95.00%]				100%	
AUC					0.66		0.64	
**Model fit**								
AIC	8197.30		8194.10		1491.90		1467.70	
BIC	8212.60		8244.80		1502.10		1513.50	

* *p* < 0.05; ** *p* < 0.01; *** *p* < 0.001.

**Table 4:** Predicted mean value of DHL for all 30 strata ranked from the lowest to the highest stratum rank.

Stratum Rank	Gender	Educational Attainment	Generation	*n* Stratum Size	Predicted Mean Value of DHL (Range: 11–55)	Approximate 95% CI
1 (Lowest)	Male	Basic	Generation Z	11	38.80	[37.00–40.60]
2	Male	Basic	Generation X	30	38.90	[37.40–40.30]
3	Male	Basic	Baby Boomer	22	39.00	[37.50–40.50]
4	Female	Basic	Generation Z	5	39.20	[37.40–40.90]
5	Male	Basic	Traditionalists	12	39.30	[37.50–41.00]
6	Female	Basic	Generation X	24	39.60	[38.10–41.10]
7	Female	Basic	Baby Boomer	26	39.70	[38.20–41.20]
8	Male	Basic	Generation Y	13	39.90	[38.40–41.50]
9	Female	Basic	Traditionalists	16	40.00	[38.30–41.80]
10	Female	Basic	Generation Y	17	40.30	[38.70–41.80]
11	Male	Medium	Generation X	92	40.40	[39.20–41.50]
12	Female	Medium	Generation Z	53	40.60	[39.20–42.10]
13	Male	Medium	Generation Z	46	40.70	[39.20–42.20]
14	Male	Higher	Generation Z	11	40.80	[39.20–42.40]
15	Male	Medium	Baby Boomer	84	40.90	[39.70–42.10]
16	Male	Medium	Traditionalists	38	41.10	[39.60–42.60]
17	Female	Higher	Generation Z	19	41.20	[39.50–42.80]
18	Male	Medium	Generation Y	82	41.30	[40.10–42.40]
19	Male	Higher	Baby Boomer	21	41.40	[40.00–42.80]
20	Female	Medium	Baby Boomer	88	41.40	[40.20–42.60]
21	Female	Medium	Generation X	105	41.50	[40.40–42.60]
22	Female	Medium	Traditionalists	31	41.70	[40.20–43.20]
23	Male	Higher	Generation X	48	41.80	[40.50–43.00]
24	Male	Higher	Generation Y	66	41.80	[40.50–43.00]
25	Male	Higher	Traditionalists	22	41.80	[40.10–43.40]
26	Female	Higher	Generation X	32	41.90	[40.60–43.20]
27	Female	Medium	Generation Y	83	42.00	[40.80–43.10]
28	Female	Higher	Baby Boomer	23	42.20	[40.80–43.70]
29	Female	Higher	Generation Y	72	42.40	[41.30–43.70]
30 (Highest)	Female	Higher	Traditionalists	8	42.50	[40.80–44.20]

Note: Stratum sample sizes (*n*) are reported alongside each predicted estimate. Approximate 95% confidence intervals overlap substantially across adjacent ranks, including between the highest-ranked stratum (*n* = 8) and its neighbors. The ranking is descriptive and exploratory and should not be interpreted as evidence of statistically significant differences between strata unless formally tested.

**Table 5:** Predicted probabilities of DHA use for all 30 strata ranked from the lowest to the highest stratum rank.

Stratum Rank	Gender	Educational Attainment	Generation	*n* Stratum Size	Predicted Probabilities of DHA Use (%)	Approximate 95% CI
1 (Lowest)	Male	Basic	Baby Boomer	22	15.50	[9.40–21.50]
2	Male	Basic	Traditionalists	12	16.40	[9.10–23.70]
3	Female	Basic	Baby Boomer	26	16.60	[10.20–22.90]
4	Female	Basic	Traditionalists	16	17.60	[9.70–25.40]
5	Male	Basic	Generation X	30	18.50	[11.90–25.10]
6	Female	Basic	Generation X	24	19.80	[12.90–26.70]
7	Male	Medium	Baby Boomer	84	21.80	[16.10–27.40]
8	Male	Medium	Traditionalists	38	23.00	[15.20–30.80]
9	Female	Medium	Baby Boomer	88	23.20	[17.40–29.00]
10	Female	Medium	Traditionalists	31	24.50	[16.20–32.80]
11	Male	Medium	Generation X	92	25.70	[20.10–31.30]
12	Female	Medium	Generation X	105	27.30	[21.50–33.10]
13	Male	Higher	Baby Boomer	21	27.70	[20.00–35.50]
14	Male	Higher	Traditionalists	22	29.20	[19.50–38.90]
15	Female	Higher	Baby Boomer	23	29.40	[21.40–37.40]
16	Female	Higher	Traditionalists	8	30.90	[20.60–41.10]
17	Male	Basic	Generation Y	13	31.60	[22.10–41.10]
18	Male	Higher	Generation X	48	32.30	[24.90–39.60]
19	Male	Basic	Generation Z	11	33.30	[22.20–44.40]
20	Female	Basic	Generation Y	17	33.40	[23.70–43.10]
21	Female	Higher	Generation X	32	34.10	[26.40–41.80]
22	Female	Basic	Generation Z	5	35.20	[23.90–46.40]
23	Male	Medium	Generation Y	82	41.30	[34.40–48.30]
24	Male	Medium	Generation Z	46	43.20	[34.30–52.20]
25	Female	Medium	Generation Y	83	43.30	[36.40–50.20]
26	Female	Medium	Generation Z	53	45.20	[36.40–54.10]
27	Male	Higher	Generation Y	66	49.20	[41.80–56.60]
28	Male	Higher	Generation Z	11	51.20	[40.80–61.50]
29	Female	Higher	Generation Y	72	51.30	[43.80–58.70]
30 (Highest)	Female	Higher	Generation Z	19	53.20	[42.90–63.50]

Note: Stratum sample sizes (*n*) are reported alongside each predicted estimate. Approximate 95% confidence intervals overlap substantially across adjacent ranks. The ranking is descriptive and exploratory and should not be interpreted as evidence of statistically significant differences between strata unless formally tested.

## Data Availability

The data are available on request due to data protection reasons.

## Supplementary Materials

The following supporting information can be downloaded at: https://www.mdpi.com/article/10.3390/healthcare14213750/s1, Table S1: Full text items for measuring digital health literacy (DHL); Table S2: Suitability of the DHL scale data for factor analysis; Table S3: Overview of item-level KMO values; Figure S1: Component loadings from the principal component analysis; Figure S2: Scree plot of the parallel analysis for the DHL items; Table S4: Stratum-level random effects (intercepts) for DHL, for the 30 strata defined by gender, educational attainment, and generation; Figure S3: Stratum-level random effects (intercepts) for DHL, for the 30 strata defined by gender, educational attainment, and generation; Figure S4: Visual model checks (posterior predictive check and influential observations) for the adjusted linear model of DHL, for the 30 strata defined by gender, educational attainment, and generation; Table S5: Stratum-level random effects (intercepts) for DHA use, for the 30 strata defined by gender, educational attainment, and generation; Figure S5: Stratum-level random effects (intercepts) for DHA use, for the 30 strata defined by gender, educational attainment, and generation; Figure S6: Visual model checks (posterior predictive check and binned residuals) for the adjusted logistic model of DHA use, for the 30 strata defined by gender, educational attainment, and generation; Table S6: Size and distribution of intersectional strata defined by gender, educational attainment, and generation; Table S7: Sample description (*N* = 1187); Table S8: Stratum-level random effects (intercepts) for DHL, for the 28 strata defined by gender, educational attainment, and generation; Figure S7: Stratum-level random effects (intercepts) for DHL, for the 28 strata defined by gender, educational attainment, and generation; Table S9: Stratum-level random effects (intercepts) for DHA use, for the 28 strata defined by gender, educational attainment, and generation; Figure S8: Stratum-level random effects (intercepts) for DHA use, for the 28 strata defined by gender, educational attainment, and generation; Table S10: Parameter estimates for linear models of DHL and logistic models of DHA use; Table S11: Predicted mean value of DHL for all 28 strata ranked from the lowest to the highest stratum rank; Figure S9: Predicted mean value of digital health literacy for all 28 strata ranked from the lowest to the highest stratum rank; Table S12: Predicted probabilities of DHA use for all 28 strata ranked from the lowest to the highest stratum rank; Figure S10: Predicted probabilities of DHA use for all 28 strata ranked from the lowest to the highest stratum rank; Table S13: Size and distribution of intersectional strata defined by gender, educational attainment, and age groups; Table S14: Sample description (*N* = 1200); Table S15: Stratum-level random effects (intercepts) for DHL, for the 30 strata defined by gender, educational attainment, and age classification (18–29 years, 30–39 years, 40–49 years, 50–59 years, and 60–74 years); Figure S11: Stratum-level random effects (intercepts) for DHL, for the 30 strata defined by gender, educational attainment, and age classification (18–29 years, 30–39 years, 40–49 years, 50–59 years, and 60–74 years); Table S16: Stratum-level random effects (intercepts) for DHA use, for the 30 strata defined by gender, educational attainment, and age classification (18–29 years, 30–39 years, 40–49 years, 50–59 years, and 60–74 years); Figure S12: Stratum-level random effects (intercepts) for DHA use, for the 30 strata defined by gender, educational attainment, and age classification (18–29 years, 30–39 years, 40–49 years, 50–59 years, and 60–74 years); Table S17: Parameter estimates for linear models of DHL and logistic models of DHA use; Table S18: Predicted mean value of DHL for all 30 strata ranked from the lowest to the highest stratum rank; Figure S13: Predicted mean value of digital health literacy for all 30 strata ranked from the lowest to the highest stratum rank; Table S19: Predicted probabilities of DHA use for all 30 strata ranked from the lowest to the highest stratum rank; Figure S14: Predicted probabilities of DHA use for all 30 strata ranked from the lowest to the highest stratum rank; File S1: R code for the MAIHDA of digital health literacy and digital health application use.

## Abbreviations

AIC	Akaike information criterion
AUC	Area under the receiver operating characteristic curve
BIC	Bayesian information criterion
CASMIN	Comparative analysis of social mobility in industrial nations
CI	Confidence interval
Cont.	Continuous variable
DHA	Digital health application(s)
DHL	Digital health literacy
DigComp 2.1	Digital competence framework for citizens 2.1
HINTS	Health Information National Trends Survey
ICC	Intraclass correlation coefficient
ID	Identifier of the relevant intersectional strata
MAIHDA	Multilevel analysis of individual heterogeneity and discriminatory accuracy
*N*	Sample size
*n*	Size of the relevant categories and intersectional strata
OR	Odds ratio
*p*	*p*-value
PCV	Proportional change in variance
Ref.	Reference category
SD	Standard deviation
SDOH	Social determinants of health
STELLAR	Online survey regarding the present and future use of digital health applications and the current degree of knowledge of digital health applications offered in Germany
VPC	Variance partition coefficient
