# Supplementary material for: Intersectionality in Digital Health Literacy and Digital Health Application Use in Germany: A MAIHDA Study by Gender, Educational Attainment, and Generation
Source: Healthcare (Basel). 2026 Nov 3;14(21):3750. doi: 10.3390/healthcare14213750 (PMC13763517; doi:10.3390/healthcare14213750)
Supplement: Supplementary file 1 [file healthcare-14-03750-s001.zip › File_S1_R_code_MAIHDA.pdf]

File S1: R code for the MAIHDA of digital health literacy and digital health application use

R code for the MAIHDA of digital health literacy

```
#Library----
library(modelbased)
library(insight)
library(datawizard)
library(parameters)
library(performance)
library(lme4)
library(haven)
library(marginaleffects)
library(psych)
library(nFactors)
library(sjPlot)
library(ggplot2)
library(DT)
library(htmltools)
library(ggpubr)

#McDonaldOmega & PCA----
dat_mcdonald <- read_sav("placeholder")
tab_om <- omega(dat_mcdonald)
print(tab_om)
txt <- capture.output(print(tab_om))

tab_componets <- datatable(principal_components(dat_mcdonald, n = "auto", rotation = "varimax"))
print(tab_componets)

check_factorstructure(dat_mcdonald)

fa.parallel(dat_mcdonald, fa = "pc")

#Load .sav file and Label----
data_MAIHDA <- read_sav("placeholder")

#recode and rename
data_MAIHDA <- datawizard::to_factor(data_MAIHDA,
                                     select = c("sex", "education", "generation"))

levels(data_MAIHDA$sex)
levels(data_MAIHDA$education)
levels(data_MAIHDA$generation)
data_MAIHDA <- data_MAIHDA |>
  datawizard::data_rename(select = "sex", replacement = "gender") |>
  datawizard::data_modify(
    gender = forcats::fct_recode(gender,
                                 "male" = "Männlich", "female" = "Weiblich"),
    education = forcats::fct_recode(education,
                                    "basic" = "niedrig", "medium" = "mittel", "high" = "hoch"),
    generation = forcats::fct_recode(generation,
```

```
                                     "traditionalist" = "Trad", "baby boomer" = "BB",
                                     "generation X" = "X", "generation Y" = "Y", "generation Z" = "Z"),
    DHL_score = as.numeric(DHL_score))


#Formation of the Strata----
data_MAIHDA$strata <- ifelse(
  is.na(data_MAIHDA$gender) | is.na(data_MAIHDA$education) | is.na(data_MAIHDA$generation),
  NA_character_,
  paste0(data_MAIHDA$gender, ", ", data_MAIHDA$education, ", ", data_MAIHDA$generation))
data_MAIHDA$strata <- factor(data_MAIHDA$strata)
data_tabulate(data_MAIHDA$strata)

#null_model----
m_null <- lmer(DHL_score ~ 1 + (1 | gender:education:generation), data = data_MAIHDA)
model_parameters(m_null)
#iccnull
icc(m_null)

#Partially-adjusted intersectional model and PCV----
m_gender <- lmer(DHL_score ~ gender + (1 | gender:education:generation), data = data_MAIHDA)
m_education <- lmer(DHL_score ~ education + (1 | gender:education:generation), data = data_MAIHDA)
m_generation <- lmer(DHL_score ~ generation + (1 | gender:education:generation), data = data_MAIHDA)

compare_parameters(m_gender, m_education, m_generation)

#ICC Attenuation
icc(m_gender)$ICC_adjusted
icc(m_education)$ICC_adjusted
icc(m_generation)$ICC_adjusted

#PCV----
v_null <- get_variance(m_null)
v_gender <- get_variance(m_gender)
v_education <- get_variance(m_education)
v_generation <- get_variance(m_generation)
# from null-model to gender-model
(v_null$var.random - v_gender$var.random) / v_null$var.random
# PCV from null-model to education-model
(v_null$var.random - v_education$var.random) / v_null$var.random
# PCV from null-model to generation-model
(v_null$var.random - v_generation$var.random) / v_null$var.random


#Full Model----
m_full <- lmer(
  DHL_score ~ gender + education + generation + (1 | gender:education:generation),
  data = data_MAIHDA)
model_parameters(m_full)
v_full <- get_variance(m_full)
(v_null$var.random - v_full$var.random) / v_null$var.random
```

```
#ICC----
#iccnull
set.seed(123)
icc(m_null,
    tolerance = 1e-05,
    ci = .95,
    iterations = 1000,
    ci_method = NULL)

#iccfull
set.seed(123)
icc(m_full,
    tolerance = 1e-05,
    ci = 0.95,
    iterations = 1000,
    ci_method = NULL)

#bootstrapped PCV----
.compute_random_vars <- function(
    model,
    tolerance,
    components = c("var.fixed", "var.random", "var.residual"),
    name_fun = "icc()",
    name_full = "ICC",
    null_model = NULL,
    model_component = NULL,
    approximation = "lognormal",
    verbose = TRUE
) {
  vars <- tryCatch(
    insight::get_variance(
      model,
      name_fun = name_fun,
      name_full = name_full,
      tolerance = tolerance,
      null_model = null_model,
      approximation = approximation,
      model_component = model_component,
      verbose = verbose
    ),
    error = function(e) {
      if (inherits(e, c("simpleError", "error")) && verbose) {
        insight::print_color(e$message, "red")
        cat("\n")
      }
      NULL
    }
  )

  if (is.null(vars) || all(is.na(vars))) {
```

```
    return(NA)
  }

  # check if we have successfully computed all variance components...
  check_elements <- sapply(components, function(.i) !is.null(vars[[.i]]))

  if (!all(check_elements)) {
    return(NA)
  }

  vars
}


# bootstrapping using "lme4::bootMer"
.boot_icc_fun_lme4 <- function(model) {
  vars <- .compute_random_vars(
    model,
    tolerance = 1e-10,
    verbose = FALSE
  )
  if (is.null(vars) || all(is.na(vars))) {
    return(NA)
  }
  vars$var.random
}


# prepare arguments for "lme4::bootMer"
.do_lme4_bootmer <- function(model, iterations, dots) {
  my_args <- list(
    model,
    .boot_icc_fun_lme4,
    nsim = iterations,
    type = "parametric",
    parallel = "no",
    use.u = FALSE,
    ncpus = 1
  )
  # add/overwrite dot-args
  if (!is.null(dots[["use.u"]])) {
    my_args$use.u <- dots[["use.u"]]
  }
  if (!is.null(dots[["re.form"]])) {
    my_args$re.form <- dots[["re.form"]]
  }
  if (!is.null(dots[["type"]])) {
    my_args$type <- dots[["type"]]
    if (my_args$type == "semiparametric") {
      my_args$use.u <- TRUE
    }
```

```
  }
  if (!is.null(dots[["parallel"]])) {
    my_args$parallel <- dots[["parallel"]]
  }
  if (!is.null(dots[["ncpus"]])) {
    my_args$ncpus <- dots[["ncpus"]]
  }
  # bootsrap
  do.call(lme4::bootMer, args = my_args)
}


n_sims <- 1000

result_full <- .do_lme4_bootmer(
  m_full,
  iterations = n_sims,
  dots = list(use.u = TRUE)
)
result_null <- .do_lme4_bootmer(
  m_null,
  iterations = n_sims,
  dots = list(use.u = TRUE)
)

out_full <- as.data.frame(result_full$t)
out_null <- as.data.frame(result_null$t)

pcvs <- (result_null$t - result_full$t) / result_null$t
pcvs <- pcvs[!is.na(pcvs) & !is.infinite(pcvs) & pcvs >= 0]

out <- ci(pcvs, method = "BCI")

cbind(
  PCV_center = (out$CI_high - out$CI_low) / 2,
  PCV_mean = mean(pcvs),
  ci(pcvs, method = "BCI"))

# investigate random effects parameters null
random_parameters(m_null)

# investigate random effects parameters full
random_parameters(m_full)

#prepredictions nullmodel
predictions_null <- estimate_means(
  m_null,
  by = c("gender", "education", "generation"),
  estimate = "average")
plot(predictions_null)
```

```r
#prepredictions fullmodel
predictions_full <- estimate_means(
  m_full,
  by = c("gender", "education", "generation"),
  estimate = "average")
plot(predictions_full)

#estimate Strata----
strata_residuals <- estimate_grouplevel(m_full)
strata_residuals
plot(strata_residuals)
shapiro.test(strata_residuals$Coefficient)
skewness(strata_residuals$Coefficient)
kurtosis(strata_residuals$Coefficient)
dt_strata_residuals <- datatable(strata_residuals)
print(dt_strata_residuals)

#visualization
#Table like evans et al - full model
tab_evans <- sjPlot::tab_model(m_full, p.style = "stars")
#Table strata model full
dt_strata <- datatable(predictions_full,options = list(order = list(list(4, "asc"))))
print(tab_evans)
print(dt_strata)

# Caterpillar
# stratum in full
predictions_full$stratum <- paste(predictions_full$gender,
                                  predictions_full$education,
                                  predictions_full$generation, sep = " | ")
predictions_full$rank <- rank(predictions_full$Mean, ties.method = "first")

# Caterpillar
ggplot(predictions_full, aes(x = rank, y = Mean)) +
  geom_hline(yintercept = mean(predictions_full$Mean), color = "red", linewidth = 1) +
  geom_pointrange(aes(ymin = CI_low, ymax = CI_high), size = 0.6) +
  geom_point(size = 2) +
  geom_text(aes(label = stratum), hjust = -0.2, size = 3, angle = -90, nudge_x = -0.4) +
  annotate("text", x = max(predictions_full$rank), y = mean(predictions_full$Mean),
           label = round(mean(predictions_full$Mean), digits = 2), vjust = 1.2, hjust = 0.25, color = "red", size = 3.5) +
  scale_x_continuous(breaks = range(predictions_full$rank),
                     labels = c("Lowest perceived\nDHL", "Highest perceived\nDHL")) +
  coord_cartesian(clip = "off") +
  ylab("Predicted mean of DHL\n(full model)") +
  xlab("stratum rank") +
  theme_bw() +
  theme(axis.text.x = element_text(size = 10),
        axis.ticks.x = element_blank(),
        plot.margin = margin(t = 10, r = 80, b = 20, l = 10))
```

```
ggqqplot(residuals(m_full), color = "black", xlab = "Theoretical Quantiles",
         ylab = "Sample Quantiles")


#compare
compare_performance(m_null, m_full)
AIC(logLik(m_null, REML = FALSE))
AIC(logLik(m_full, REML = FALSE))

check_convergence(m_null)
check_convergence(m_full)
check_singularity(m_null)
check_singularity(m_full)

#browsable
browsable(tagList(tags$pre(paste(txt, collapse = "\n"))))
browsable(tagList(HTML(tab_evans$knitr)))
browsable(tagList(dt_strata))

pdf("log30check.pdf", width = 10, height = 15, pointsize = 10)
check_model(m_full)
dev.off()
```

R code for the MAIHDA of digital health application use

```
#Library----
library(modelbased)
library(insight)
library(datawizard)
library(parameters)
library(performance)
library(lme4)
library(haven)
library(marginaleffects)
library(psych)
library(nFactors)
library(sjPlot)
library(ggplot2)
library(DT)
library(htmltools)
library(ggpubr)

#load .sav file and label----
data_MAIHDA <- read_sav("placeholder")

#recode and rename
data_MAIHDA <- datawizard::to_factor(data_MAIHDA,
                                     select = c("sex", "education", "generation"))

levels(data_MAIHDA$sex)
levels(data_MAIHDA$education)
```

```
levels(data_MAIHDA$generation)
data_MAIHDA <- data_MAIHDA |>
  datawizard::data_rename(select = "sex", replacement = "gender") |>
  datawizard::data_modify(
    gender = forcats::fct_recode(gender,
                                 "male" = "Männlich", "female" = "Weiblich"),
    education = forcats::fct_recode(education,
                                    "basic" = "niedrig", "medium" = "mittel", "high" = "hoch"),
    generation = forcats::fct_recode(generation,
                                     "traditionalist" = "Trad", "baby boomer" = "BB",
                                     "generation X" = "X", "generation Y" = "Y", "generation Z" = "Z"))

#Formation of the Strata----
data_MAIHDA$strata <- ifelse(
  is.na(data_MAIHDA$gender) | is.na(data_MAIHDA$education) | is.na(data_MAIHDA$generation),
  NA_character_,
  paste0(data_MAIHDA$gender, ", ", data_MAIHDA$education, ", ", data_MAIHDA$generation))
data_MAIHDA$strata <- factor(data_MAIHDA$strata)
data_tabulate(data_MAIHDA$strata)

#null_model----
m_null <- glmer(use_corr ~ 1 + (1 | gender:education:generation),family = binomial(link = "logit") , data = data_MAIHDA)
model_parameters(m_null)
#iccnull
icc(m_null)

#Partially-adjusted intersectional model and PCV----
m_gender <- glmer(use_corr ~ gender + (1 | gender:education:generation),family = binomial(link = "logit"), data = data_MAIHDA)
m_education <- glmer(use_corr ~ education + (1 | gender:education:generation),family = binomial(link = "logit"), data = data_MAIHDA)
m_generation <- glmer(use_corr ~ generation + (1 | gender:education:generation),family = binomial(link = "logit"), data = data_MAIHDA)

compare_parameters(m_gender, m_education, m_generation)

#ICC Attenuation
icc(m_gender)$ICC_adjusted
icc(m_education)$ICC_adjusted
icc(m_generation)$ICC_adjusted

#PCV----
v_null <- get_variance(m_null)
v_gender <- get_variance(m_gender)
v_education <- get_variance(m_education)
v_generation <- get_variance(m_generation)
# from null-model to gender-model
(v_null$var.random - v_gender$var.random) / v_null$var.random
# PCV from null-model to education-model
(v_null$var.random - v_education$var.random) / v_null$var.random
```

```r
# PCV from null-model to generation-model
(v_null$var.random - v_generation$var.random) / v_null$var.random


#Full Model----
m_full <- glmer(
 use_corr ~ gender + education + generation + (1 | gender:education:generation),family = binomial(link = "logit") ,
  data = data_MAIHDA)
v_full <- get_variance(m_full)
(v_null$var.random - v_full$var.random) / v_null$var.random

#ICC----
#iccnull
set.seed(123)
icc(m_null,
    tolerance = 1e-05,
    ci = .95,
    iterations = 1000,
    ci_method = NULL)

#iccfull
set.seed(123)
tictoc::tic()
icc(m_full,
    tolerance = 1e-05,
    ci = 0.95,
    iterations = 1000,
    ci_method = NULL)
tictoc::toc()


tictoc::tic()
#bootstrapped PCV----
.compute_random_vars <- function(
    model,
    tolerance,
    components = c("var.fixed", "var.random", "var.residual"),
    name_fun = "icc()",
    name_full = "ICC",
    null_model = NULL,
    model_component = NULL,
    approximation = "lognormal",
    verbose = TRUE
) {
  vars <- tryCatch(
    insight::get_variance(
      model,
      name_fun = name_fun,
      name_full = name_full,
      tolerance = tolerance,
      null_model = null_model,
```

```
      approximation = approximation,
      model_component = model_component,
      verbose = verbose
    ),
    error = function(e) {
      if (inherits(e, c("simpleError", "error")) && verbose) {
        insight::print_color(e$message, "red")
        cat("\n")
      }
      NULL
    }
  )

  if (is.null(vars) || all(is.na(vars))) {
    return(NA)
  }

  # check if we have successfully computed all variance components...
  check_elements <- sapply(components, function(.i) !is.null(vars[[.i]]))

  if (!all(check_elements)) {
    return(NA)
  }

  vars
}


# bootstrapping using "lme4::bootMer"
.boot_icc_fun_lme4 <- function(model) {
  vars <- .compute_random_vars(
    model,
    tolerance = 1e-10,
    verbose = FALSE
  )
  if (is.null(vars) || all(is.na(vars))) {
    return(NA)
  }
  vars$var.random
}


# prepare arguments for "lme4::bootMer"
.do_lme4_bootmer <- function(model, iterations, dots) {
  my_args <- list(
    model,
    .boot_icc_fun_lme4,
    nsim = iterations,
    type = "parametric",
    parallel = "no",
    use.u = TRUE,
```

```
    ncpus = 1
  )
  # add/overwrite dot-args
  if (!is.null(dots[["use.u"]])) {
    my_args$use.u <- dots[["use.u"]]
  }
  if (!is.null(dots[["re.form"]])) {
    my_args$re.form <- dots[["re.form"]]
  }
  if (!is.null(dots[["type"]])) {
    my_args$type <- dots[["type"]]
    if (my_args$type == "semiparametric") {
      my_args$use.u <- TRUE
    }
  }
  if (!is.null(dots[["parallel"]])) {
    my_args$parallel <- dots[["parallel"]]
  }
  if (!is.null(dots[["ncpus"]])) {
    my_args$ncpus <- dots[["ncpus"]]
  }
  # bootsrap
  do.call(lme4::bootMer, args = my_args)
}


n_sims <- 1000

result_full <- .do_lme4_bootmer(
  m_full,
  iterations = n_sims,
  dots = list(use.u = TRUE)
)
result_null <- .do_lme4_bootmer(
  m_null,
  iterations = n_sims,
  dots = list(use.u = TRUE)
)

out_full <- as.data.frame(result_full$t)
out_null <- as.data.frame(result_null$t)

pcvs <- (result_null$t - result_full$t) / result_null$t
pcvs <- pcvs[!is.na(pcvs) & !is.infinite(pcvs) & pcvs >= 0]

out <- ci(pcvs, method = "BCI")

cbind(
  PCV_center = (out$CI_high - out$CI_low) / 2,
  PCV_mean = mean(pcvs),
  ci(pcvs, method = "BCI"))
```

```r
tictoc::toc()


#AUC
performance_roc(m_null)
performance_roc(m_full)
plot(performance_roc(m_null, m_full))

roc <- performance_roc(m_null)
bayestestR::area_under_curve(roc$Specificity, roc$Sensitivity)

# investigate random effects parameters null
random_parameters(m_null)

# investigate random effects parameters full
random_parameters(m_full)

#prepredictions nullmodel
predictions_null <- estimate_means(
  m_null,
  by = c("gender", "education", "generation"),
  estimate = "average")
plot(predictions_null)

#prepredictions fullmodel
predictions_full <- estimate_means(
  m_full,
  by = c("gender", "education", "generation"),
  estimate = "average")
plot(predictions_full)

#estimate Strata----
strata_residuals <- estimate_grouplevel(m_full)
strata_residuals
plot(strata_residuals)
shapiro.test(strata_residuals$Coefficient)
skewness(strata_residuals$Coefficient)
kurtosis(strata_residuals$Coefficient)
dt_strata_residuals <- datatable(strata_residuals)
print(dt_strata_residuals)

#visualization
#Table like evans et al - full model
tab_evans <- sjPlot::tab_model(m_full, p.style = "stars")
tab_evans_null <- sjPlot::tab_model(m_null, p.style = "stars")
#Table strata model full
dt_strata <- datatable(predictions_full,options = list(order = list(list(4, "asc"))))
print(tab_evans)
print(tab_evans_null)
print(dt_strata)
```

```
predictions_full |>
  select(gender, education, generation, Probability, CI_low, CI_high) |>
  arrange(desc(Probability)) |>
  gt() |>
  fmt_number(columns = c(Probability, CI_low, CI_high), decimals = 3)



# Caterpillar
predictions_full$stratum <- paste(predictions_full$gender,
                                  predictions_full$education,
                                  predictions_full$generation, sep = " | ")
predictions_full$rank <- rank(predictions_full$Probability, ties.method = "first")

ggplot(predictions_full, aes(x = rank, y = Probability)) +
  geom_hline(yintercept = mean(predictions_full$Probability), color = "red", linewidth = 1) +
  geom_pointrange(aes(ymin = CI_low, ymax = CI_high), size = 0.6) +
  geom_point(size = 2) +
  geom_text(aes(label = stratum), hjust = 0.6, size = 3, angle = -90, nudge_x = -0.4) +
  annotate("text", x = max(predictions_full$rank), y = mean(predictions_full$Probability),
           label = scales::percent(mean(predictions_full$Probability), accuracy = 1),
           vjust = 1.2, hjust = 0.25, color = "red", size = 3.5) +
  scale_x_continuous(breaks = range(predictions_full$rank),
                     labels = c("Lowest probability\nof use", "Highest probability\nof use")) +
  coord_cartesian(clip = "off") +
  ylab("Predicted probability of use\n(full model)") +
  xlab("stratum rank") +
  theme_bw() +
  theme(axis.text.x = element_text(size = 10),
        axis.ticks.x = element_blank(),
        plot.margin = margin(t = 10, r = 80, b = 20, l = 10))

#compare
compare_performance(m_null, m_full)
AIC(logLik(m_null, REML = FALSE))
AIC(logLik(m_full, REML = FALSE))

ggqqplot(residuals(m_full), color = "black", xlab = "Theoretical Quantiles",
         ylab = "Sample Quantiles")

check_convergence(m_null)
check_convergence(m_full)
check_singularity(m_null)
check_singularity(m_full)


performance_mor(m_full)

#browsable
browsable(tagList(HTML(tab_evans$knitr)))
```

```
browsable(tagList(dt_strata))


pdf("log30check.pdf", width = 10, height = 15, pointsize = 10)
check_model(m_full)
dev.off()

model_parameters(m_full)
check_normality(m_full, effects = "random")
```
