# Supplementary material for: Intersectionality in Digital Health Literacy and Digital Health Application Use in Germany: A MAIHDA Study by Gender, Educational Attainment, and Generation
Source: Healthcare (Basel). 2026 Nov 3;14(21):3750. doi: 10.3390/healthcare14213750 (PMC13763517; doi:10.3390/healthcare14213750)

healthcare

MDPI

Table S1. Full text items for measuring digital health literacy (DHL)

	Please indicate the extent to which you agree with each of the following statements.					
**Item**		Fully agree	Agree	Neutral	Disagree	Fully disagree
**DHL_1**	I have access to the digital devices (e.g., a computer or laptop) that I need to obtain and use health-related information.	◎ 5	◎ 4	◎ 3	◎ 2	◎ 1
**DHL_2**	I am able to search for health-related information in digital media efficiently and purposefully.	◎ 5	◎ 4	◎ 3	◎ 2	◎ 1
**DHL_3**	I am able to deal effectively with the amount of health-related information I find in digital media.	◎ 5	◎ 4	◎ 3	◎ 2	◎ 1
**DHL_4**	I can quickly identify which health-related information is relevant to me.	◎ 5	◎ 4	◎ 3	◎ 2	◎ 1

**DHL_5**	I actively engage with the technologies behind digital health information and services.	◎ 5	◎ 4	◎ 3	◎ 2	◎ 1
**DHL_6**	I am able to assess the opportunities and limitations of digital media for obtaining and using health-related information.	◎ 5	◎ 4	◎ 3	◎ 2	◎ 1
**DHL_7**	When using digital media to obtain health-related information, I feel safe and in control.	◎ 5	◎ 4	◎ 3	◎ 2	◎ 1
**DHL_8**	It is easy for me to find digital health services (e.g., apps) that meet my needs.	◎ 5	◎ 4	◎ 3	◎ 2	◎ 1
**DHL_9**	I can critically evaluate health-related information that I find online.	◎ 5	◎ 4	◎ 3	◎ 2	◎ 1
**DHL_10**	I can distinguish reliable health-related information from unreliable information found online.	◎ 5	◎ 4	◎ 3	◎ 2	◎ 1

**DHL_11**	Before using a digital health service, I check how my personal data will be collected, used, and protected.	◎ 5	◎ 4	◎ 3	◎ 2	◎ 1

Table S2. Suitability of the DHL scale data for factor analysis

Test	Statistic	Value
Bartlett's test of sphericity	$\chi^2(55)$	5753.08, $p < 0.001$
Kaiser-Meyer-Olkin (KMO) measure of sampling adequacy, overall	MSA	0.94

Table S3. Overview of item-level KMO values

DHL item	Individual KMO (MSA)
DHL_1	0.95
DHL_2	0.94
DHL_3	0.94
DHL_4	0.94
DHL_5	0.88
DHL_6	0.94
DHL_7	0.93
DHL_8	0.94
DHL_9	0.95
DHL_10	0.93
DHL_11	0.94

Figure S1. Component loadings from the principal component analysis

Variable	PC1	Complexity	Uniqueness	MSA
DHL_1	0.57	1.00	0.68	0.95
DHL_2	0.80	1.00	0.36	0.94
DHL_3	0.80	1.00	0.36	0.94
DHL_4	0.80	1.00	0.36	0.94
DHL_5	0.53	1.00	0.72	0.88
DHL_6	0.79	1.00	0.38	0.94
DHL_7	0.66	1.00	0.57	0.93
DHL_8	0.76	1.00	0.43	0.94
DHL_9	0.66	1.00	0.56	0.95
DHL_10	0.76	1.00	0.43	0.93
DHL_11	0.51	1.00	0.74	0.94

Figure S2. Scree plot of the parallel analysis for the DHL items

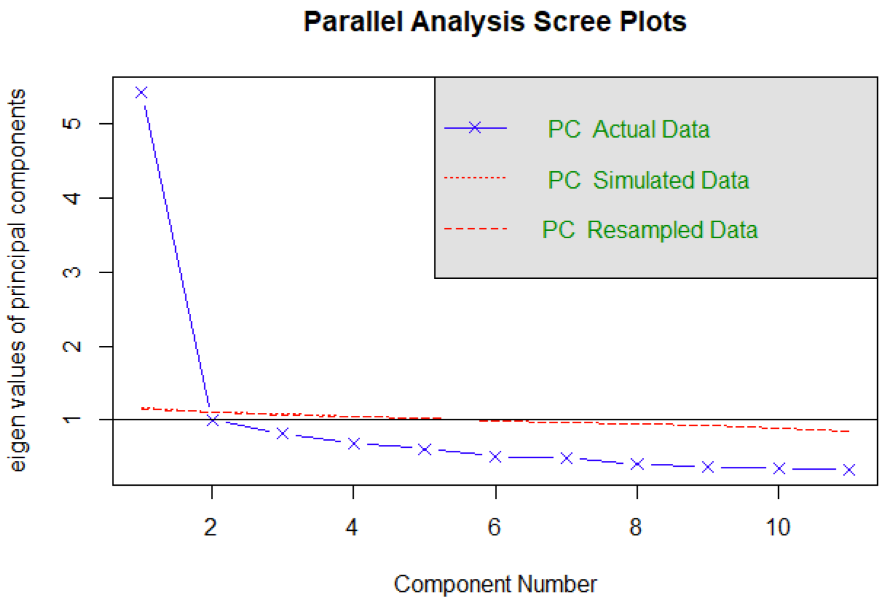

Table S4. Stratum-level random effects (intercepts) for DHL, for the 30 strata defined by gender, educational attainment, and

g

> estimate_grouplevel(m_full)

Group	Level	Parameter	Coefficient	SE	95% CI
gender:education:generation	female:basic:baby boomer	(Intercept)	-0.02	0.50	[-1.01, 0.96]
gender:education:generation	female:basic:generation X	(Intercept)	-0.01	0.51	[-1.01, 0.98]
gender:education:generation	female:basic:generation Y	(Intercept)	0.04	0.51	[-0.97, 1.04]
gender:education:generation	female:basic:generation Z	(Intercept)	6.62e-03	0.53	[-1.03, 1.05]
gender:education:generation	female:basic:traditionalist	(Intercept)	-1.25e-03	0.52	[-1.01, 1.01]
gender:education:generation	female:high:baby boomer	(Intercept)	0.21	0.51	[-0.78, 1.21]
gender:education:generation	female:high:generation X	(Intercept)	-0.04	0.50	[-1.01, 0.93]
gender:education:generation	female:high:generation Y	(Intercept)	-0.06	0.46	[-0.96, 0.83]
gender:education:generation	female:high:generation Z	(Intercept)	-0.30	0.51	[-1.30, 0.71]
gender:education:generation	female:high:traditionalist	(Intercept)	0.17	0.53	[-0.86, 1.21]
gender:education:generation	female:medium:baby boomer	(Intercept)	-0.01	0.44	[-0.88, 0.86]
gender:education:generation	female:medium:generation X	(Intercept)	0.20	0.43	[-0.65, 1.04]
gender:education:generation	female:medium:generation Y	(Intercept)	0.03	0.45	[-0.84, 0.91]
gender:education:generation	female:medium:generation Z	(Intercept)	-0.21	0.47	[-1.14, 0.72]
gender:education:generation	female:medium:traditionalist	(Intercept)	-8.37e-04	0.50	[-0.98, 0.97]
gender:education:generation	male:basic:baby boomer	(Intercept)	-0.14	0.51	[-1.14, 0.85]
gender:education:generation	male:basic:generation X	(Intercept)	-0.16	0.50	[-1.14, 0.82]
gender:education:generation	male:basic:generation Y	(Intercept)	0.29	0.52	[-0.73, 1.30]
gender:education:generation	male:basic:generation Z	(Intercept)	0.19	0.52	[-0.83, 1.22]
gender:education:generation	male:basic:traditionalist	(Intercept)	-0.18	0.52	[-1.20, 0.84]
gender:education:generation	male:high:baby boomer	(Intercept)	-0.10	0.51	[-1.10, 0.90]
gender:education:generation	male:high:generation X	(Intercept)	0.39	0.48	[-0.55, 1.33]
gender:education:generation	male:high:generation Y	(Intercept)	-0.21	0.46	[-1.12, 0.69]
gender:education:generation	male:high:generation Z	(Intercept)	-0.08	0.52	[-1.10, 0.94]
gender:education:generation	male:high:traditionalist	(Intercept)	0.02	0.51	[-0.98, 1.01]
gender:education:generation	male:medium:baby boomer	(Intercept)	0.06	0.45	[-0.81, 0.94]
gender:education:generation	male:medium:generation X	(Intercept)	-0.38	0.44	[-1.24, 0.49]
gender:education:generation	male:medium:generation Y	(Intercept)	-0.08	0.45	[-0.96, 0.80]
gender:education:generation	male:medium:generation Z	(Intercept)	0.39	0.48	[-0.56, 1.33]
gender:education:generation	male:medium:traditionalist	(Intercept)	-7.24e-03	0.49	[-0.97, 0.95]

Figure S3. Stratum-level random effects (intercepts) for DHL, for the 30 strata defined by gender, educational attainment, and generation

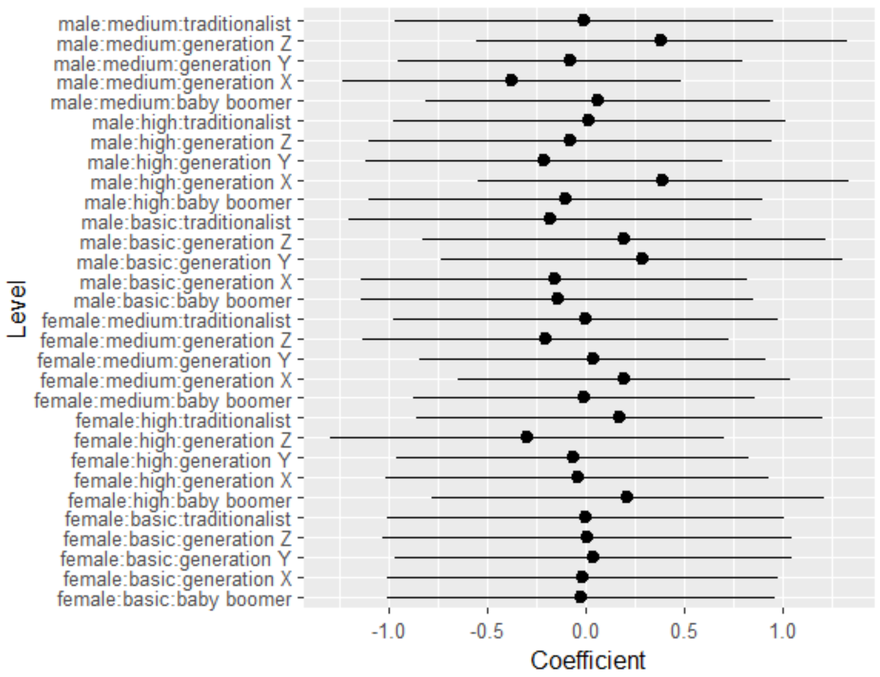

Figure S4. Visual model checks (posterior predictive check and influen-tial observations) for the adjusted linear model of DHL, for the 30 strata defined by gender, educational attainment, and generation

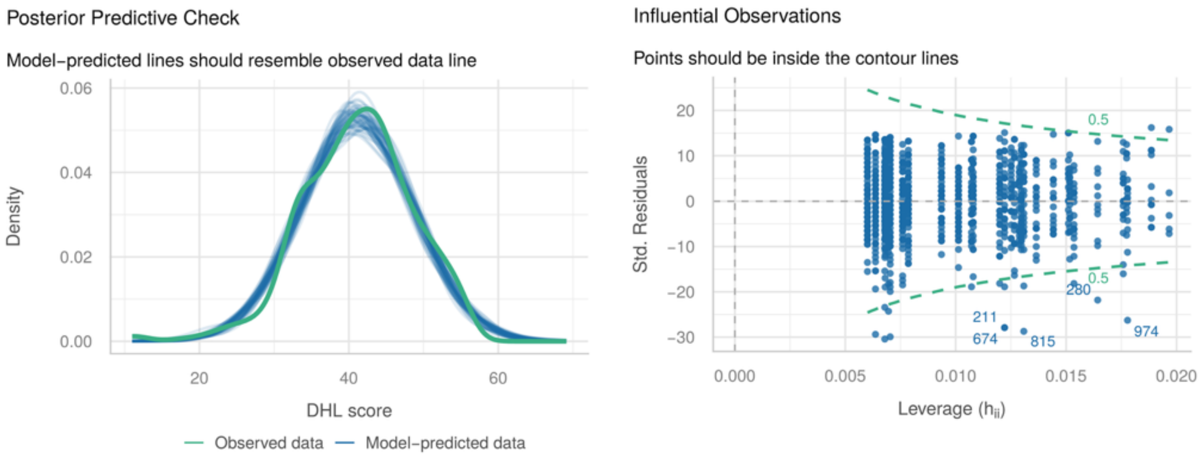

Table S5. Stratum-level random effects (intercepts) for DHA use, for the 30 strata defined by gender, educational attainment, and

Group	Level	Parameter	Coefficient	SE	95% CI
gender:education:generation	female:basic:baby boomer	(Intercept)	0	0	[0.00, 0.00]
gender:education:generation	female:basic:generation X	(Intercept)	0	0	[0.00, 0.00]
gender:education:generation	female:basic:generation Y	(Intercept)	0	0	[0.00, 0.00]
gender:education:generation	female:basic:generation Z	(Intercept)	0	0	[0.00, 0.00]
gender:education:generation	female:basic:traditionalist	(Intercept)	0	0	[0.00, 0.00]
gender:education:generation	female:high:baby boomer	(Intercept)	0	0	[0.00, 0.00]
gender:education:generation	female:high:generation X	(Intercept)	0	0	[0.00, 0.00]
gender:education:generation	female:high:generation Y	(Intercept)	0	0	[0.00, 0.00]
gender:education:generation	female:high:generation Z	(Intercept)	0	0	[0.00, 0.00]
gender:education:generation	female:high:traditionalist	(Intercept)	0	0	[0.00, 0.00]
gender:education:generation	female:medium:baby boomer	(Intercept)	0	0	[0.00, 0.00]
gender:education:generation	female:medium:generation X	(Intercept)	0	0	[0.00, 0.00]
gender:education:generation	female:medium:generation Y	(Intercept)	0	0	[0.00, 0.00]
gender:education:generation	female:medium:generation Z	(Intercept)	0	0	[0.00, 0.00]
gender:education:generation	female:medium:traditionalist	(Intercept)	0	0	[0.00, 0.00]
gender:education:generation	male:basic:baby boomer	(Intercept)	0	0	[0.00, 0.00]
gender:education:generation	male:basic:generation X	(Intercept)	0	0	[0.00, 0.00]
gender:education:generation	male:basic:generation Y	(Intercept)	0	0	[0.00, 0.00]
gender:education:generation	male:basic:generation Z	(Intercept)	0	0	[0.00, 0.00]
gender:education:generation	male:basic:traditionalist	(Intercept)	0	0	[0.00, 0.00]
gender:education:generation	male:high:baby boomer	(Intercept)	0	0	[0.00, 0.00]
gender:education:generation	male:high:generation X	(Intercept)	0	0	[0.00, 0.00]
gender:education:generation	male:high:generation Y	(Intercept)	0	0	[0.00, 0.00]
gender:education:generation	male:high:generation Z	(Intercept)	0	0	[0.00, 0.00]
gender:education:generation	male:high:traditionalist	(Intercept)	0	0	[0.00, 0.00]
gender:education:generation	male:medium:baby boomer	(Intercept)	0	0	[0.00, 0.00]
gender:education:generation	male:medium:generation X	(Intercept)	0	0	[0.00, 0.00]
gender:education:generation	male:medium:generation Y	(Intercept)	0	0	[0.00, 0.00]
gender:education:generation	male:medium:generation Z	(Intercept)	0	0	[0.00, 0.00]
gender:education:generation	male:medium:traditionalist	(Intercept)	0	0	[0.00, 0.00]

Figure S5. Stratum-level random effects (intercepts) for DHA use, for the 30 strata defined by gender, educational attainment, and

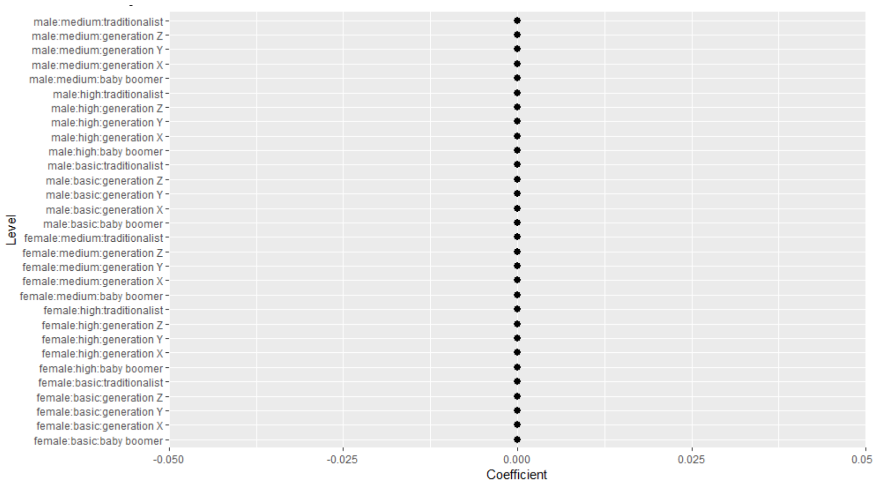

Figure S6. Visual model checks (posterior predictive check and binned residuals) for the adjusted logistic model of DHA use, for the 30 strata defined by gender, educational attainment, and generation

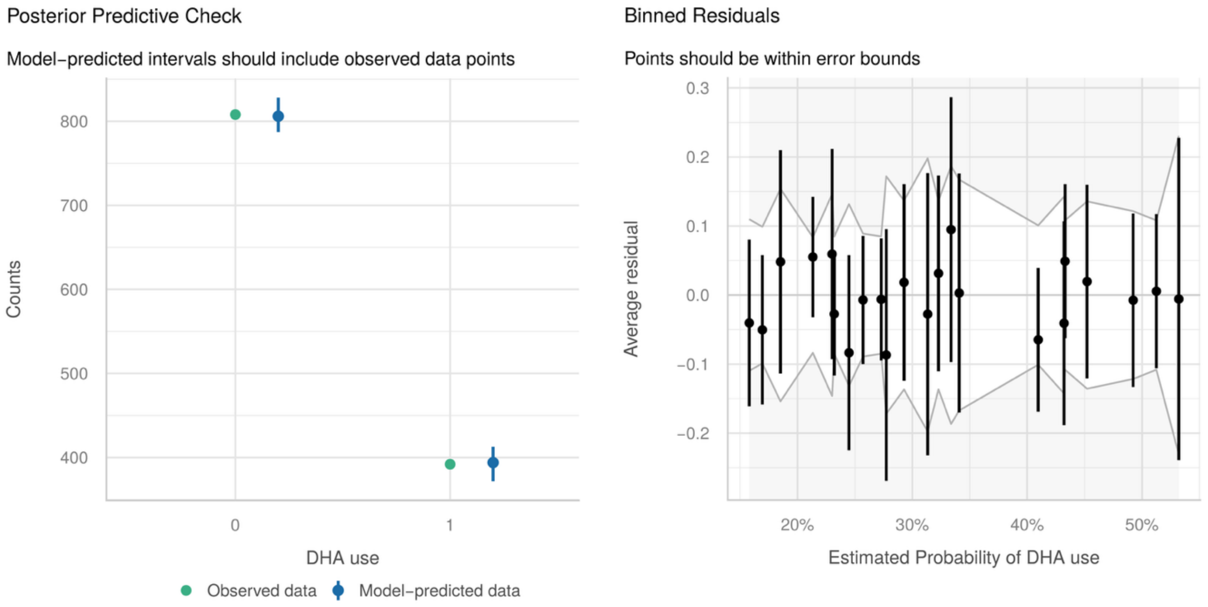

Sensitivity Analysis. 28 intersectional strata by gender, educational attainment, and generation

Table S6. Size and distribution of intersectional strata defined by gender, educa- tional attainment, and generation

Size per stratum	n	%
100 or more individuals	1	3.60
50 or more individuals	9	32.10
30 or more individuals	15	53.60
20 or more individuals	21	75.00
10 or more individuals	28	100.00
Fewer than 10 individuals	0	0.00

Table S7. Sample description (N = 1,187)

	n	%
**Gender**		
Female	589	49.6
Male	598	50.4
**Generation** (46.7[a]; 15.2[b])		
Traditionalists (Year of birth: 1922–1955) (70.3[a]; 2.1[b]; Min. 67, Max. 74[c])	119	10.0
Baby Boomer (Year of birth: 1956–1965) (61.1[a]; 2.7[b]; Min. 57, Max. 66[c])	264	22.2
Generation X (Year of birth: 1966–1980) (49.8[a]; 4.5[b]; Min. 42, Max. 56[c])	331	27.9
Generation Y (Year of birth: 1981–1995) (34.0[a]; 4.4[b]; Min. 27, Max. 41[c])	333	28.1

Generation Z (Year of birth: 1996–present)<br>(22.9[a]; 2.5[b]; Min. 18, Max. 26[c])	140	11.8
**Educational attainment**		
Basic	171	14.4
Medium	702	59.1
Higher	314	26.5
**Outcome variables**		
Use of digital health applications (DHA use)	387	32.6
Digital health literacy (cont.) (DHL)	41.1[d]; 7.3[b]	-

[a] Mean age; [b] Standard deviation; [c] Range; [d] Mean value

Table S8. Stratum-level random effects (intercepts) for DHL, for the 28 strata defined by gender, educational attainment, and generation

Group	Level	Parameter	Coefficient	SE	95% CI
gender:education:generation	female:basic:baby boomer	(Intercept)	-0.02	0.52	[-1.05, 1.00]
gender:education:generation	female:basic:generation X	(Intercept)	-0.01	0.52	[-1.04, 1.01]
gender:education:generation	female:basic:generation Y	(Intercept)	0.04	0.53	[-1.01, 1.08]
gender:education:generation	female:basic:traditionalist	(Intercept)	0.03	0.54	[-1.02, 1.08]
gender:education:generation	female:high:baby boomer	(Intercept)	0.25	0.53	[-0.78, 1.28]
gender:education:generation	female:high:generation X	(Intercept)	-0.02	0.51	[-1.02, 0.99]
gender:education:generation	female:high:generation Y	(Intercept)	-0.03	0.47	[-0.95, 0.89]
gender:education:generation	female:high:generation Z	(Intercept)	-0.30	0.53	[-1.35, 0.74]
gender:education:generation	female:medium:baby boomer	(Intercept)	-5.28e-03	0.45	[-0.90, 0.89]
gender:education:generation	female:medium:generation X	(Intercept)	0.21	0.44	[-0.65, 1.07]
gender:education:generation	female:medium:generation Y	(Intercept)	0.03	0.46	[-0.87, 0.93]
gender:education:generation	female:medium:generation Z	(Intercept)	-0.21	0.49	[-1.17, 0.74]
gender:education:generation	female:medium:traditionalist	(Intercept)	0.05	0.52	[-0.96, 1.06]
gender:education:generation	male:basic:baby boomer	(Intercept)	-0.16	0.53	[-1.20, 0.87]
gender:education:generation	male:basic:generation X	(Intercept)	-0.19	0.52	[-1.20, 0.83]
gender:education:generation	male:basic:generation Y	(Intercept)	0.30	0.54	[-0.76, 1.36]
gender:education:generation	male:basic:generation Z	(Intercept)	0.20	0.54	[-0.86, 1.27]
gender:education:generation	male:basic:traditionalist	(Intercept)	-0.18	0.54	[-1.24, 0.88]
gender:education:generation	male:high:baby boomer	(Intercept)	-0.10	0.53	[-1.14, 0.94]
gender:education:generation	male:high:generation X	(Intercept)	0.44	0.49	[-0.53, 1.41]
gender:education:generation	male:high:generation Y	(Intercept)	-0.21	0.48	[-1.15, 0.72]
gender:education:generation	male:high:generation Z	(Intercept)	-0.08	0.54	[-1.15, 0.98]
gender:education:generation	male:high:traditionalist	(Intercept)	0.06	0.53	[-0.97, 1.10]
gender:education:generation	male:medium:baby boomer	(Intercept)	0.04	0.46	[-0.86, 0.94]
gender:education:generation	male:medium:generation X	(Intercept)	-0.43	0.45	[-1.31, 0.46]
gender:education:generation	male:medium:generation Y	(Intercept)	-0.12	0.46	[-1.02, 0.78]
gender:education:generation	male:medium:generation Z	(Intercept)	0.40	0.50	[-0.58, 1.37]
gender:education:generation	male:medium:traditionalist	(Intercept)	0.04	0.51	[-0.96, 1.03]

Figure S7. Stratum-level random effects (intercepts) for DHL, for the 28 strata de- fined by gender, educational attainment, and generation

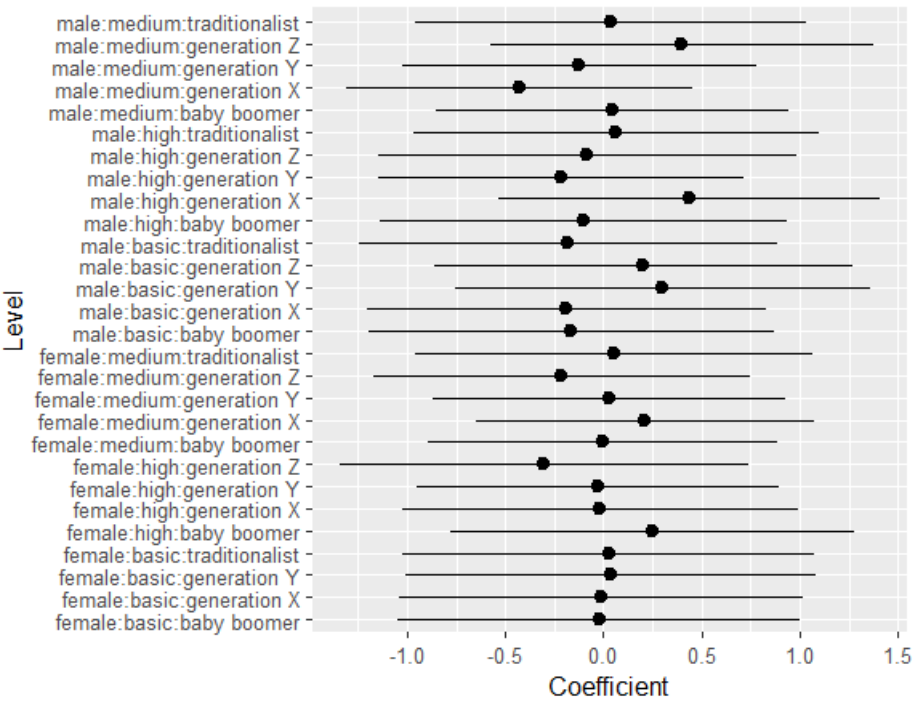

Table S9. Stratum-level random effects (intercepts) for DHA use, for the 28 strata defined by gender, educational attainment, and generation

Group	Level	Parameter	Coefficient	SE	95% CI
gender:education:generation	female:basic:baby boomer	(Intercept)	0	0	[0.00, 0.00]
gender:education:generation	female:basic:generation X	(Intercept)	0	0	[0.00, 0.00]
gender:education:generation	female:basic:generation Y	(Intercept)	0	0	[0.00, 0.00]
gender:education:generation	female:basic:traditionalist	(Intercept)	0	0	[0.00, 0.00]
gender:education:generation	female:high:baby boomer	(Intercept)	0	0	[0.00, 0.00]
gender:education:generation	female:high:generation X	(Intercept)	0	0	[0.00, 0.00]
gender:education:generation	female:high:generation Y	(Intercept)	0	0	[0.00, 0.00]
gender:education:generation	female:high:generation Z	(Intercept)	0	0	[0.00, 0.00]
gender:education:generation	female:medium:baby boomer	(Intercept)	0	0	[0.00, 0.00]
gender:education:generation	female:medium:generation X	(Intercept)	0	0	[0.00, 0.00]
gender:education:generation	female:medium:generation Y	(Intercept)	0	0	[0.00, 0.00]
gender:education:generation	female:medium:generation Z	(Intercept)	0	0	[0.00, 0.00]
gender:education:generation	female:medium:traditionalist	(Intercept)	0	0	[0.00, 0.00]
gender:education:generation	male:basic:baby boomer	(Intercept)	0	0	[0.00, 0.00]
gender:education:generation	male:basic:generation X	(Intercept)	0	0	[0.00, 0.00]
gender:education:generation	male:basic:generation Y	(Intercept)	0	0	[0.00, 0.00]
gender:education:generation	male:basic:generation Z	(Intercept)	0	0	[0.00, 0.00]
gender:education:generation	male:basic:traditionalist	(Intercept)	0	0	[0.00, 0.00]
gender:education:generation	male:high:baby boomer	(Intercept)	0	0	[0.00, 0.00]
gender:education:generation	male:high:generation X	(Intercept)	0	0	[0.00, 0.00]
gender:education:generation	male:high:generation Y	(Intercept)	0	0	[0.00, 0.00]
gender:education:generation	male:high:generation Z	(Intercept)	0	0	[0.00, 0.00]
gender:education:generation	male:high:traditionalist	(Intercept)	0	0	[0.00, 0.00]
gender:education:generation	male:medium:baby boomer	(Intercept)	0	0	[0.00, 0.00]
gender:education:generation	male:medium:generation X	(Intercept)	0	0	[0.00, 0.00]
gender:education:generation	male:medium:generation Y	(Intercept)	0	0	[0.00, 0.00]
gender:education:generation	male:medium:generation Z	(Intercept)	0	0	[0.00, 0.00]
gender:education:generation	male:medium:traditionalist	(Intercept)	0	0	[0.00, 0.00]

Figure S8. Stratum-level random effects (intercepts) for DHA use, for the 28 strata defined by gender, educational attainment, and generation

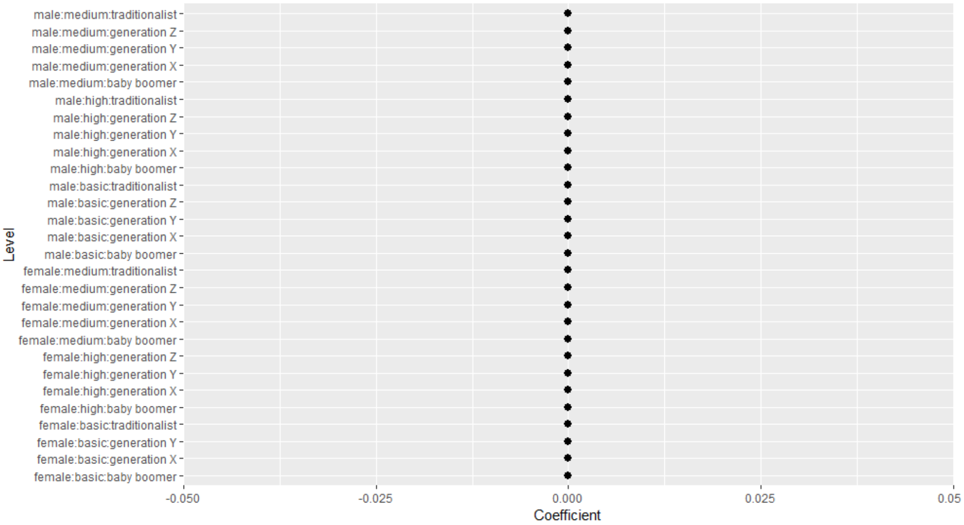

Table S10. Parameter estimates for linear models of DHL and logistic models of DHA use

	Digital health literacy				Digital health application use			
	Linear Model 1A		Linear Model 1B		Logistic Model 1A		Logistic Model 1B	
	Estimate	[95% CI]	Estimate	[95% CI]	OR	[95% CI]	OR	[95% CI]
**Fixed effects:** **Regression coefficients**								
Intercept	41.01***	[40.48–41.54]	39.21***	[37.46–40.97]	0.44***	[0.34-0.56]	0.19***	[0.11–0.33]
**Gender**								
Male (Ref.)			-	-			-	-
Female			0.47	[-0.50–1.44]			1.08	[0.84–1.39]
**Educational attainment**								
Basic (Ref.)			-	-			-	-
Medium			1.69*	[0.35–3.03]			1.60*	[1.06–2.42]
Higher			2.15**	[0.65–3.65]			2.22***	[1.42–3.46]
**Generation**								
Traditionalists (Ref.)			-	-			-	-

Baby Boomer			0.03	[-1.72–1.78]			0.91	[0.55–1.52]
Generation X			-0.07	[-1.77–1.63]			1.13	[0.69–1.85]
Generation Y			0.57	[-1.15–2.29]			2.29***	[1.41–3.72]
Generation Z			-0.55	[-2.51–1.41]			2.39**	[1.38–4.12]
**Random effects:**<br>**Variance**								
Residual variance (σ^2)	53.20<br>(SD 7.30)		53.30<br>(SD 7.30)		3.29<br>(SD 1.81)		3.29<br>(SD 1.81)	
Stratum-level variance (τ_{00})	0.62<br>(SD 0.78)		0.31<br>(SD 0.56)		0.24<br>(SD 0.49)		0.00	
VPC [95% CI]	1.10%<br>[0.07-3.50%]		0.60%<br>[0.02-3.00%]		6.90%<br>[6.20-16.40%]		0.00%	
PCV [95% CI]			48.50%<br>[4.50-96.50%]				100%	
AUC					0.66		0.64	
**Model fit**								
AIC	8103.00		8104.90		1474.10		1450.40	
BIC	8118.20		8155.70		1484.20		1496.10	

* $p < 0.05$; ** $p < 0.01$; *** $p < 0.001$.

healthcare

MDPI

Table S11. Predicted mean value of DHL for all 28 strata ranked from the lowest to the highest stratum rank

Stratum rank	Gender	Educational attainment	Generation	n Stratum size	Predicted mean value of DHL (Range: 11–55)	Approximate 95% CI
1 (Lowest)	Male	Basic	Generation Z	11	38.90	[37.00-40.70]
2	Male	Basic	Generation X	30	39.00	[37.50-40.40]
3	Male	Basic	Traditionalists	12	39.00	[37.30-40.80]
4	Male	Basic	Baby Boomer	22	39.10	[37.60-40.60]
5	Female	Basic	Generation X	24	39.60	[38.20-41.10]
6	Female	Basic	Baby Boomer	26	39.70	[38.20-41.20]
7	Female	Basic	Traditionalists	16	39.70	[37.90-41.50]
8	Male	Basic	Generation Y	13	40.10	[38.50-41.60]
9	Female	Basic	Generation Y	17	40.30	[38.70-41.80]
10	Male	Medium	Generation X	92	40.40	[39.30-41.60]
11	Female	Medium	Generation Z	53	40.60	[39.20-42.10]
12	Male	Higher	Generation Z	11	40.70	[39.10-42.40]
13	Male	Medium	Generation Z	46	40.80	[39.30-42.20]
14	Male	Medium	Traditionalists	38	40.90	[39.40-42.50]
15	Male	Medium	Baby Boomer	84	41.00	[39.80-42.20]
16	Female	Higher	Generation Z	19	41.00	[39.30-42.60]
17	Male	Higher	Baby Boomer	21	41.30	[39.90-42.70]
18	Male	Medium	Generation Y	82	41.40	[40.20-42.50]
19	Female	Medium	Baby Boomer	88	41.40	[40.20-42.60]
20	Female	Medium	Traditionalists	31	41.40	[39.80-43.00]
21	Male	Higher	Traditionalists	22	41.40	[39.70-43.10]

22	Female	Medium	Generation X	105	41.50	[40.40-42.70]
23	Male	Higher	Generation Y	66	41.70	[40.50-42.90]
24	Male	Higher	Generation X	48	41.70	[40.40-43.00]
25	Female	Higher	Generation X	32	41.70	[40.40-43.10]
26	Female	Medium	Generation Y	83	42.00	[40.80-43.10]
27	Female	Higher	Baby Boomer	23	42.10	[40.70-43.60]
28 (Highest)	Female	Higher	Generation Y	72	42.40	[41.10-43.60]

Figure S9. Predicted mean value of digital health literacy for all 28 strata ranked from the lowest to the highest stratum rank

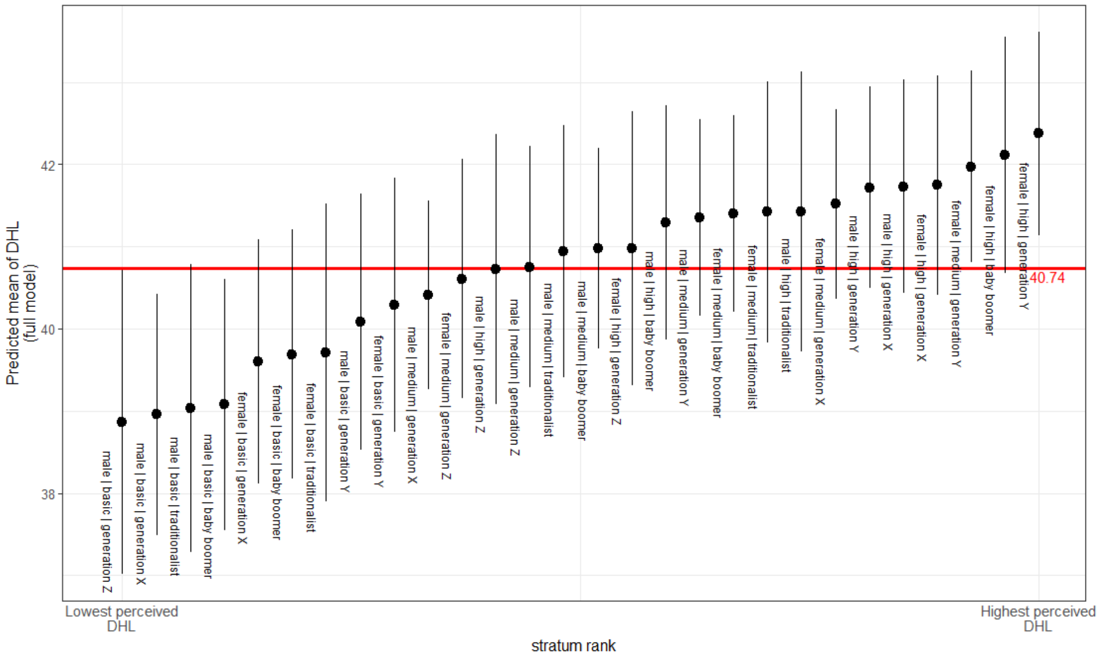

Table S12. Predicted probabilities of DHA use for all 28 strata ranked from the low- est to the highest stratum rank

**Stratum rank**	**Gender**	**Educational attainment**	**Generation**	**n Stratum size**	**Predicted proba-bilities of DHA use (%)**	**Approximate 95% CI**
1 (Lowest)	Male	Basic	Baby Boomer	22	14.90	[9.00–20.90]

2	Female	Basic	Baby Boomer	26	15.90	[9.70–22.20]
3	Male	Basic	Traditionalists	12	16.20	[8.80–23.60]
4	Female	Basic	Traditionalists	16	17.20	[9.20–25.20]
5	Male	Basic	Generation X	30	17.90	[11.40–24.40]
6	Female	Basic	Generation X	24	19.00	[12.20–25.90]
7	Male	Medium	Baby Boomer	84	21.90	[16.20–27.60]
8	Female	Medium	Baby Boomer	88	23.30	[17.50–29.10]
9	Male	Medium	Traditionalists	38	23.60	[15.50–31.70]
10	Female	Medium	Traditionalists	31	25.00	[16.30–33.70]
11	Male	Medium	Generation X	92	25.80	[20.20–31.50]
12	Female	Medium	Generation X	105	27.30	[21.50–33.10]
13	Male	Higher	Baby Boomer	21	28.00	[20.20–35.80]
14	Female	Higher	Baby Boomer	23	29.60	[21.50–37.70]
15	Male	Higher	Traditionalists	22	29.90	[19.70–40.20]
16	Male	Basic	Generation Y	13	30.60	[21.10–40.20]
17	Male	Basic	Generation Z	11	31.50	[20.40–42.70]
18	Female	Basic	Generation Y	17	32.30	[22.50–42.10]
19	Male	Higher	Generation X	48	32.50	[25.10–40.00]
20	Female	Higher	Generation X	32	34.20	[26.50–42.00]
21	Male	Medium	Generation Y	82	41.40	[34.40–48.40]
22	Male	Medium	Generation Z	46	42.40	[33.40–51.50]
23	Female	Medium	Generation Y	83	43.30	[36.40–50.10]
24	Female	Medium	Generation Z	53	44.30	[35.40–53.30]
25	Male	Higher	Generation Y	66	49.50	[42.00–56.90]
26	Male	Higher	Generation Z	11	50.50	[40.10–61.00]
27	Female	Higher	Generation Y	72	51.40	[43.90–58.80]
28 (Highest)	Female	Higher	Generation Z	19	52.40	[41.90–62.90]

Figure S10. Predicted probabilities of DHA use for all 28 strata ranked from the lowest to the highest stratum rank

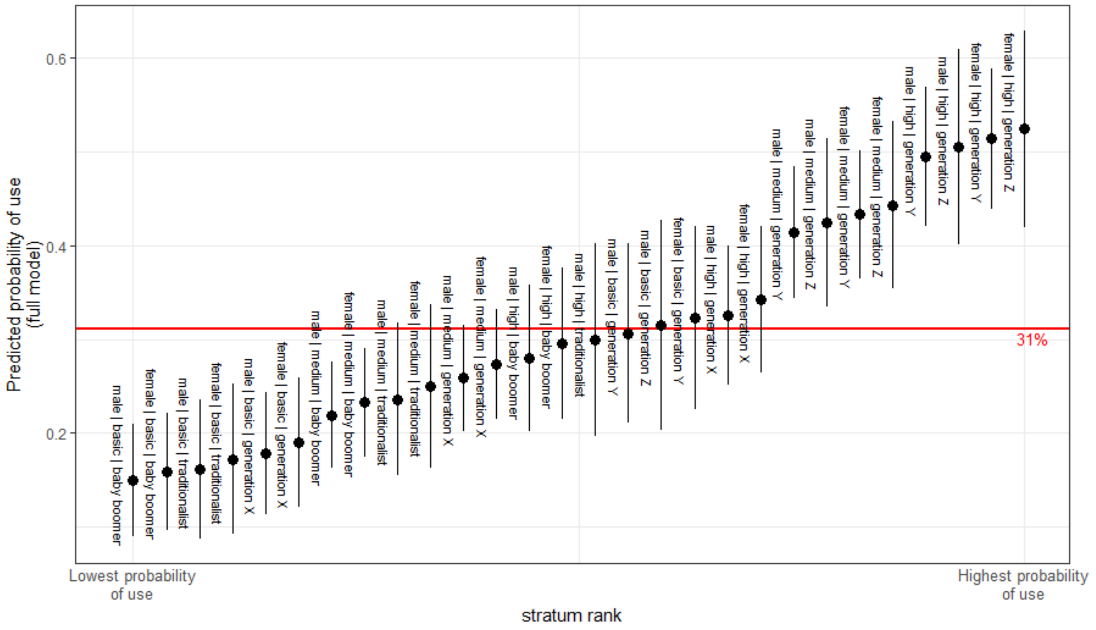

Sensitivity Analysis. Age classification (18-29 years, 30-39 years, 40-49 years, 50-59 years, and 60-74 years)

Table S13. Size and distribution of intersectional strata defined by gender, educa- tional attainment, and age groups

Size per stratum	n	%
100 or more individuals	0	0.00
50 or more individuals	10	33.30
30 or more individuals	16	53.30
20 or more individuals	23	76.70
10 or more individuals	27	90.00
Fewer than 10 individuals	3	10.00

Table S14. Sample description (N = 1,200)

	n	%
**Gender**		
Female	602	50.2
Male	598	49.8
**Age groups** (46.8[a]; 15.3[b])		
60-74 years (65.9[a]; 4.2[b])	301	25.1
50-59 years (54.9[a]; 2.9[b])	269	22.4
40-49 years (44.2[a]; 3.0[b])	204	17.0

30-39 years<br>(34.4[a]; 2.8[b])	210	17.5
18-29 years<br>(24.5[a]; 3.2[b])	216	18.0
**Educational attainment**		
Basic	176	14.7
Medium	702	58.5
Higher	322	26.8
**Outcome variables**		
Use of digital health applications (DHA use)	392	32.7
Digital health literacy (cont.) (DHL)	41.1[c]; 7.4[b]	-

[a] Mean age; [b] Standard deviation; [c] Mean value

Table S15. Stratum-level random effects (intercepts) for DHL, for the 30 strata de- fined by gender, educational attainment, and age classification (18-29 years, 30-39 years, 40-49 years, 50-59 years, and 60-74 years)

Group	Level	Parameter	Coefficient	SE	95% CI
gender:education:age_groups	female:basic:18 yrs - 29 yrs	(Intercept)	0.10	0.61	[-1.09, 1.28]
gender:education:age_groups	female:basic:30 yrs - 39 yrs	(Intercept)	-0.06	0.61	[-1.25, 1.13]
gender:education:age_groups	female:basic:40 yrs - 49 yrs	(Intercept)	0.13	0.60	[-1.05, 1.31]
gender:education:age_groups	female:basic:50 yrs - 59 yrs	(Intercept)	-0.02	0.58	[-1.15, 1.10]
gender:education:age_groups	female:basic:60 yrs - 74 yrs	(Intercept)	-0.15	0.56	[-1.25, 0.95]
gender:education:age_groups	female:high:18 yrs - 29 yrs	(Intercept)	-0.54	0.57	[-1.65, 0.58]
gender:education:age_groups	female:high:30 yrs - 39 yrs	(Intercept)	0.10	0.53	[-0.94, 1.14]
gender:education:age_groups	female:high:40 yrs - 49 yrs	(Intercept)	9.75e-03	0.57	[-1.11, 1.13]
gender:education:age_groups	female:high:50 yrs - 59 yrs	(Intercept)	-0.10	0.59	[-1.25, 1.05]
gender:education:age_groups	female:high:60 yrs - 74 yrs	(Intercept)	0.42	0.57	[-0.70, 1.54]
gender:education:age_groups	female:medium:18 yrs - 29 yrs	(Intercept)	-0.26	0.50	[-1.24, 0.72]
gender:education:age_groups	female:medium:30 yrs - 39 yrs	(Intercept)	0.19	0.55	[-0.88, 1.26]
gender:education:age_groups	female:medium:40 yrs - 49 yrs	(Intercept)	0.02	0.52	[-1.00, 1.03]
gender:education:age_groups	female:medium:50 yrs - 59 yrs	(Intercept)	0.05	0.49	[-0.90, 1.00]
gender:education:age_groups	female:medium:60 yrs - 74 yrs	(Intercept)	0.11	0.49	[-0.84, 1.07]
gender:education:age_groups	male:basic:18 yrs - 29 yrs	(Intercept)	0.40	0.60	[-0.77, 1.56]
gender:education:age_groups	male:basic:30 yrs - 39 yrs	(Intercept)	0.25	0.61	[-0.95, 1.45]
gender:education:age_groups	male:basic:40 yrs - 49 yrs	(Intercept)	-0.21	0.60	[-1.39, 0.96]
gender:education:age_groups	male:basic:50 yrs - 59 yrs	(Intercept)	0.09	0.56	[-1.02, 1.19]
gender:education:age_groups	male:basic:60 yrs - 74 yrs	(Intercept)	-0.51	0.58	[-1.64, 0.62]
gender:education:age_groups	male:high:18 yrs - 29 yrs	(Intercept)	-0.15	0.57	[-1.27, 0.96]
gender:education:age_groups	male:high:30 yrs - 39 yrs	(Intercept)	-0.19	0.55	[-1.26, 0.88]
gender:education:age_groups	male:high:40 yrs - 49 yrs	(Intercept)	0.26	0.56	[-0.84, 1.36]
gender:education:age_groups	male:high:50 yrs - 59 yrs	(Intercept)	0.17	0.57	[-0.95, 1.28]
gender:education:age_groups	male:high:60 yrs - 74 yrs	(Intercept)	0.02	0.56	[-1.07, 1.11]
gender:education:age_groups	male:medium:18 yrs - 29 yrs	(Intercept)	0.46	0.52	[-0.57, 1.48]
gender:education:age_groups	male:medium:30 yrs - 39 yrs	(Intercept)	-0.29	0.52	[-1.32, 0.73]
gender:education:age_groups	male:medium:40 yrs - 49 yrs	(Intercept)	-0.20	0.52	[-1.23, 0.82]
gender:education:age_groups	male:medium:50 yrs - 59 yrs	(Intercept)	-0.18	0.50	[-1.17, 0.80]
gender:education:age_groups	male:medium:60 yrs - 74 yrs	(Intercept)	0.11	0.48	[-0.84, 1.06]

>

Figure S11. Stratum-level random effects (intercepts) for DHL, for the 30 strata de-fined by gender, educational attainment, and age classification (18-29 years, 30-39 years, 40-49 years, 50-59 years, and 60-74 years)

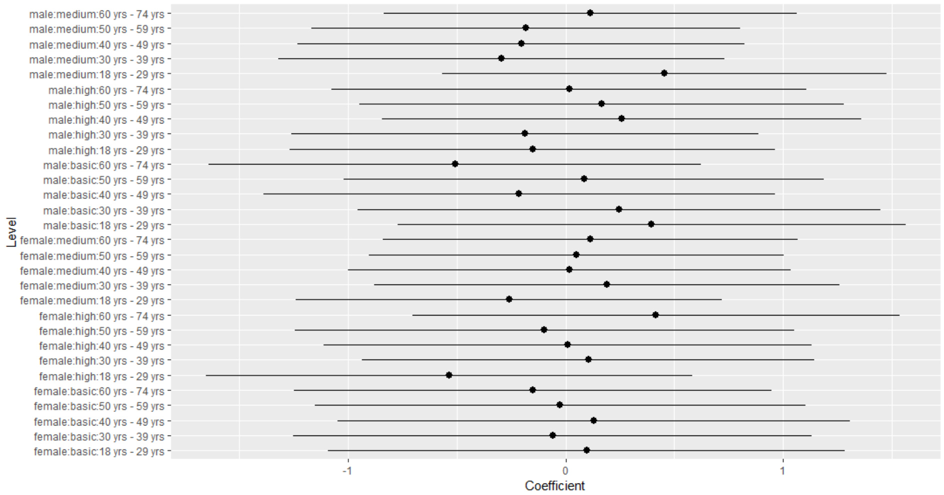

Table S16. Stratum-level random effects (intercepts) for DHA use, for the 30 strata defined by gender, educational attainment, and age classification (18-29 years, 30-39 years, 40-49 years, 50-59 years, and 60-74 years)

Group	Level	Parameter	Coefficient	SE	95% CI
gender:education:age_groups	female:basic:18 yrs - 29 yrs	(Intercept)	5.28e-03	0.09	[-0.16, 0.17]
gender:education:age_groups	female:basic:30 yrs - 39 yrs	(Intercept)	0.01	0.09	[-0.16, 0.18]
gender:education:age_groups	female:basic:40 yrs - 49 yrs	(Intercept)	2.72e-03	0.09	[-0.17, 0.17]
gender:education:age_groups	female:basic:50 yrs - 59 yrs	(Intercept)	-0.02	0.09	[-0.19, 0.15]
gender:education:age_groups	female:basic:60 yrs - 74 yrs	(Intercept)	-0.01	0.09	[-0.18, 0.15]
gender:education:age_groups	female:high:18 yrs - 29 yrs	(Intercept)	0.01	0.08	[-0.15, 0.18]
gender:education:age_groups	female:high:30 yrs - 39 yrs	(Intercept)	1.94e-03	0.08	[-0.16, 0.16]
gender:education:age_groups	female:high:40 yrs - 49 yrs	(Intercept)	0.01	0.08	[-0.15, 0.18]
gender:education:age_groups	female:high:50 yrs - 59 yrs	(Intercept)	-0.01	0.09	[-0.18, 0.16]
gender:education:age_groups	female:high:60 yrs - 74 yrs	(Intercept)	5.15e-03	0.08	[-0.16, 0.17]
gender:education:age_groups	female:medium:18 yrs - 29 yrs	(Intercept)	0.02	0.08	[-0.14, 0.18]
gender:education:age_groups	female:medium:30 yrs - 39 yrs	(Intercept)	0.02	0.08	[-0.14, 0.18]
gender:education:age_groups	female:medium:40 yrs - 49 yrs	(Intercept)	-2.24e-03	0.08	[-0.16, 0.16]
gender:education:age_groups	female:medium:50 yrs - 59 yrs	(Intercept)	1.82e-03	0.08	[-0.16, 0.16]
gender:education:age_groups	female:medium:60 yrs - 74 yrs	(Intercept)	-0.05	0.08	[-0.21, 0.11]
gender:education:age_groups	male:basic:18 yrs - 29 yrs	(Intercept)	8.75e-03	0.09	[-0.16, 0.18]
gender:education:age_groups	male:basic:30 yrs - 39 yrs	(Intercept)	1.32e-03	0.09	[-0.17, 0.17]
gender:education:age_groups	male:basic:40 yrs - 49 yrs	(Intercept)	4.12e-03	0.09	[-0.16, 0.17]
gender:education:age_groups	male:basic:50 yrs - 59 yrs	(Intercept)	0.02	0.09	[-0.15, 0.18]
gender:education:age_groups	male:basic:60 yrs - 74 yrs	(Intercept)	-0.01	0.09	[-0.18, 0.15]
gender:education:age_groups	male:high:18 yrs - 29 yrs	(Intercept)	-0.01	0.08	[-0.18, 0.15]
gender:education:age_groups	male:high:30 yrs - 39 yrs	(Intercept)	-0.02	0.08	[-0.18, 0.15]
gender:education:age_groups	male:high:40 yrs - 49 yrs	(Intercept)	2.90e-03	0.08	[-0.16, 0.17]
gender:education:age_groups	male:high:50 yrs - 59 yrs	(Intercept)	5.31e-03	0.08	[-0.16, 0.17]
gender:education:age_groups	male:high:60 yrs - 74 yrs	(Intercept)	-2.37e-03	0.08	[-0.17, 0.16]
gender:education:age_groups	male:medium:18 yrs - 29 yrs	(Intercept)	-0.04	0.08	[-0.20, 0.13]
gender:education:age_groups	male:medium:30 yrs - 39 yrs	(Intercept)	-0.02	0.08	[-0.18, 0.14]
gender:education:age_groups	male:medium:40 yrs - 49 yrs	(Intercept)	-0.02	0.08	[-0.18, 0.14]
gender:education:age_groups	male:medium:50 yrs - 59 yrs	(Intercept)	9.27e-03	0.08	[-0.15, 0.17]
gender:education:age_groups	male:medium:60 yrs - 74 yrs	(Intercept)	0.07	0.08	[-0.09, 0.23]

Figure S12. Stratum-level random effects (intercepts) for DHA use, for the 30 strata defined by gender, educational attainment, and age classification (18-29 years, 30-

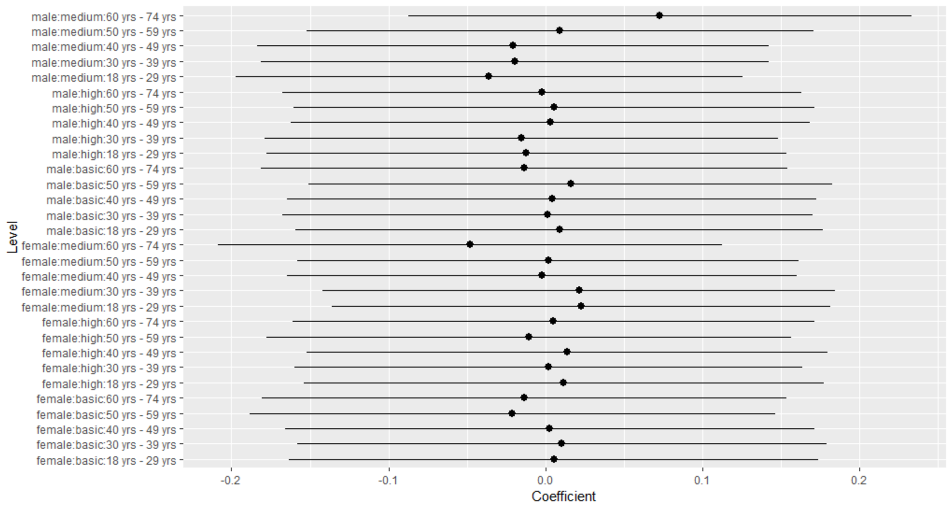

healthcare

MDPI

Table S17. Parameter estimates for linear models of DHL and logistic models of DHA use

	Digital health literacy				Digital health application use			
	Linear Model 1A		Linear Model 1B		Logistic Model 1A		Logistic Model 1B	
	Estimate	[95% CI]	Estimate	[95% CI]	OR	[95% CI]	OR	[95% CI]
**Fixed effects:**<br>**Regression coefficients**								
Intercept	40.78***	[39.96–41.61]	39.56***	[38.07–41.05]	0.47***	[0.38–0.59]	0.19***	[0.12–0.30]
**Gender**								
Male (Ref.)			-	-			-	-
Female			0.61	[-0.37–1.58]			1.10	[0.85–1.42]
**Educational attainment**								
Basic (Ref.)			-	-			-	-
Medium			1.64*	[0.28–2.99]			1.49	[0.99–2.25]
Higher			2.25**	[0.75–3.75]			2.12***	[1.36–3.30]
**Age groups**								
60-74 years (Ref.)			-	-			-	-
50-59 years			-0.73	[-2.17–0.71]			1.16	[0.78-1.74]
40-49 years			-0.10	[-1.63–1.42]			1.35	[0.88-2.06]
30-39 years			0.27	[-1.27–1.81]			2.30***	[1.52-3.46]
18-29 years			-0.95	[-2.47–0.56]			2.75***	[1.84-4.11]
**Random effects:**<br>**Variance**								
Residual variance (σ^2)	53.20 (SD 7.30)		53.30 (SD 7.30)		3.29 (SD 1.81)		3.29 (SD 1.81)	

Stratum-level variance (τ_{00})	1.10 (SD 1.05)		0.39 (SD 0.63)		0.23 (SD 0.48)		0.01 (SD 0.09)	
VPC [95% CI]	2.00% [0.20-4.50%]		0.70% [0.00-3.00%]		6.70% [1.90-11.10%]		0.20% [0.00-0.90%]	
PCV [95% CI]			64.50% [4.00-96.00%]				96.80% [76.00-100%]	
AUC					0.65		0.64	
**Model fit**								
AIC	8195.50		8195.50		1493.90		1472.50	
BIC	8210.80		8246.40		1504.10		1518.30	

* $p < 0.05$; ** $p < 0.01$; *** $p < 0.001$.

healthcare MDPI

Table S18. Predicted mean value of DHL for all 30 strata ranked from the lowest to the highest stratum rank

Stratum rank	Gender	Educational attainment	Age group	n Stratum size	Predicted mean value of DHL (Range: 11–55)	Approximate 95% CI
1 (Lowest)	Male	Basic	50–59 years	32	38.90	[37.40–40.40]
2	Male	Basic	18–29 years	14	39.00	[37.30–40.70]
3	Male	Basic	60–74 years	24	39.10	[37.60–40.50]
4	Male	Basic	40–49 years	12	39.20	[37.60–40.90]
5	Female	Basic	18–29 years	9	39.30	[37.60–41.00]
6	Female	Basic	50–59 years	25	39.40	[37.90–40.90]
7	Female	Basic	60–74 years	34	40.00	[38.50–41.50]
8	Male	Basic	30–39 years	6	40.10	[38.30–41.80]
9	Female	Basic	40–49 years	12	40.20	[38.50–41.90]
10	Male	Medium	50–59 years	75	40.30	[39.10–41.50]
11	Female	Basic	30–39 years	8	40.40	[38.70–42.10]
12	Female	Medium	18–29 years	77	40.60	[39.30–41.90]
13	Male	Medium	18–29 years	60	40.70	[39.40–42.00]
14	Male	Higher	18–29 years	28	40.70	[39.30–42.20]
15	Male	Medium	40–49 years	58	40.90	[39.60–42.20]
16	Female	Higher	18–29 years	28	40.90	[39.50–42.40]
17	Female	Medium	50–59 years	89	41.10	[39.90–42.40]
18	Male	Medium	30–39 years	58	41.20	[39.80–42.50]
19	Male	Higher	50–59 years	29	41.20	[39.80–42.70]
20	Male	Medium	60–74 years	91	41.30	[40.10–42.50]
21	Female	Higher	50–59 years	19	41.60	[40.10–43.00]

22	Female	Medium	40–49 years	62	41.70	[40.40–43.00]
23	Male	Higher	60–74 years	36	41.80	[40.50–43.20]
24	Male	Higher	30–39 years	42	41.90	[40.50–43.30]
25	Female	Medium	60–74 years	89	41.90	[40.70–43.10]
26	Male	Higher	40–49 years	33	42.00	[40.50–43.40]
27	Female	Medium	30–39 years	43	42.30	[40.90–43.60]
28	Female	Higher	40–49 years	27	42.30	[40.90–43.80]
29	Female	Higher	30–39 years	53	42.80	[41.40–44.20]
30 (Highest)	Female	Higher	60–74 years	27	42.80	[41.40–44.20]

Figure S13. Predicted mean value of digital health literacy for all 30 strata ranked from the lowest to the highest stratum rank

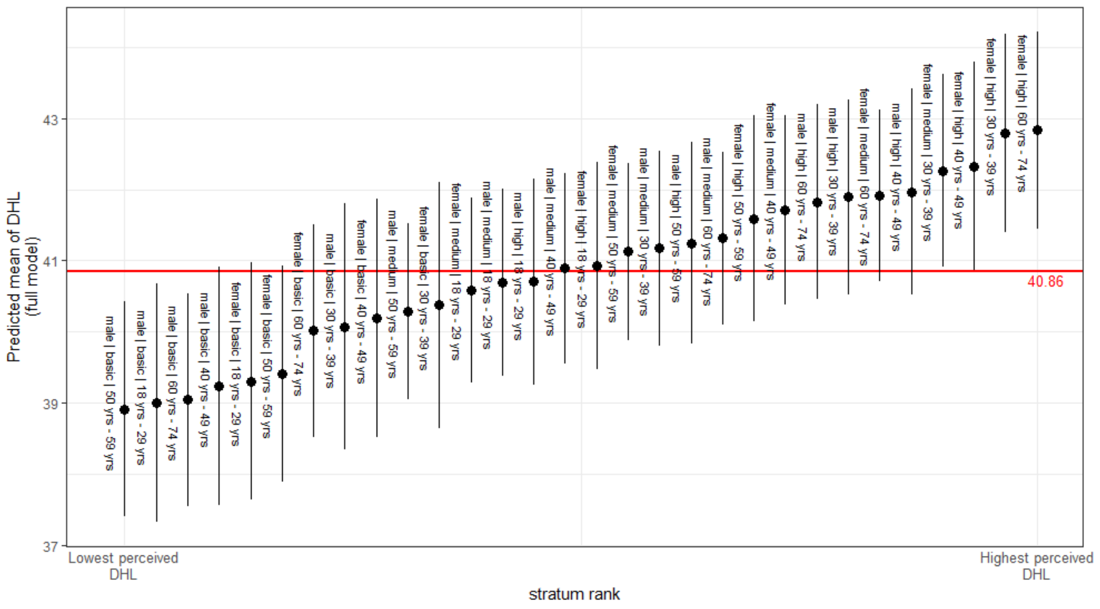

Table S19. Predicted probabilities of DHA use for all 30 strata ranked from the low- est to the highest stratum rank

Stratum rank	Gender	Educational attainment	Age group	n Stratum size	Predicted probabilities of DHA use (%)	Approximate 95% CI
1 (Lowest)	Male	Basic	60–74 years	24	15.80	[9.70–21.90]
2	Female	Basic	60–74 years	34	17.10	[10.60–23.60]
3	Male	Basic	50–59 years	32	18.40	[11.60–25.20]
4	Female	Basic	50–59 years	25	19.20	[12.20–26.30]
5	Male	Basic	40–49 years	12	20.40	[12.40–28.40]
6	Female	Basic	40–49 years	12	22.00	[13.50–30.40]
7	Female	Medium	60–74 years	89	22.90	[17.10–28.80]
8	Male	Medium	60–74 years	91	23.40	[17.40–29.40]
9	Male	Medium	50–59 years	75	25.00	[18.70–31.30]
10	Female	Medium	50–59 years	89	26.70	[20.20–33.20]
11	Male	Medium	40–49 years	58	27.20	[20.10–34.30]
12	Male	Higher	60–74 years	36	28.70	[21.00–36.30]
13	Female	Medium	40–49 years	62	29.50	[22.10–36.90]
14	Male	Basic	30–39 years	6	30.40	[20.00–40.80]
15	Female	Higher	60–74 years	27	30.80	[22.70–38.90]
16	Male	Higher	50–59 years	29	32.00	[23.60–40.40]
17	Female	Basic	30–39 years	8	32.70	[21.90–43.40]
18	Female	Higher	50–59 years	19	33.80	[25.10–42.50]
19	Male	Basic	18–29 years	14	34.50	[23.80–45.20]
20	Male	Higher	40–49 years	33	35.20	[26.40–44.00]
21	Female	Basic	18–29 years	9	36.60	[25.70–47.50]
22	Female	Higher	40–49 years	27	37.60	[28.50–46.80]

23	Male	Medium	30–39 years	58	39.00	[30.70–47.20]
24	Female	Medium	30–39 years	43	42.30	[33.90–50.70]
25	Male	Medium	18–29 years	60	43.00	[34.90–51.00]
26	Female	Medium	18–29 years	77	46.70	[38.70–54.80]
27	Male	Higher	30–39 years	42	47.70	[39.00–56.30]
28	Female	Higher	30–39 years	53	50.50	[41.70–59.30]
29	Male	Higher	18–29 years	28	52.20	[42.90–61.60]
30 (Highest)	Female	Higher	18–29 years	28	55.20	[46.00–64.40]

Figure S14. Predicted probabilities of DHA use for all 30 strata ranked from the lowest to the highest stratum rank

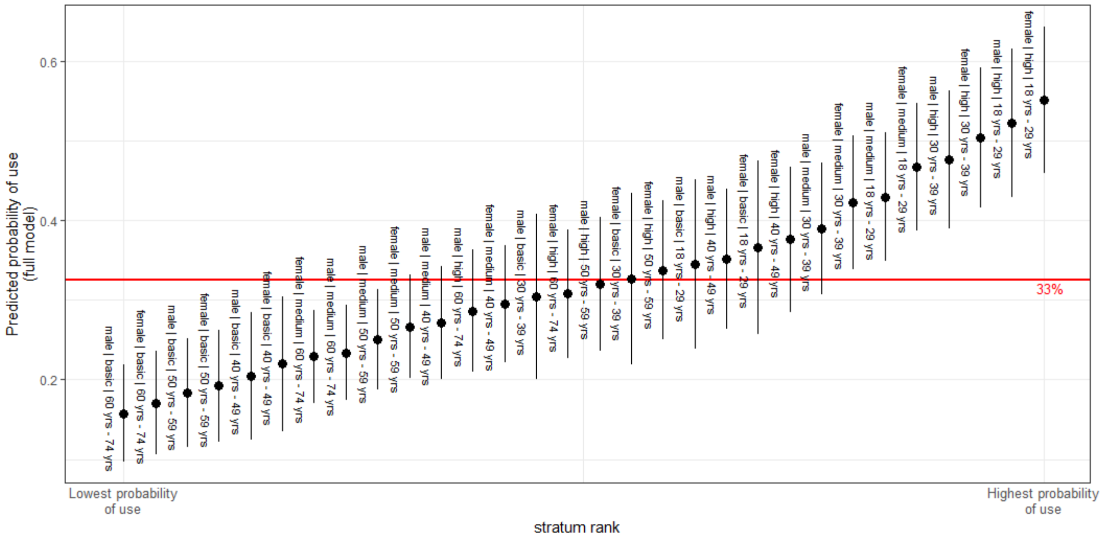

Supplement: Supplementary file 1 [file healthcare-14-03750-s001.zip › healthcare-4546992-supplementary.pdf]
